# Two Locomotor Traits Show Different Patterns of Developmental Plasticity Between Closely Related Clonal and Sexual Fish

**DOI:** 10.3389/fphys.2021.740604

**Published:** 2021-10-12

**Authors:** Kate L. Laskowski, Frank Seebacher, Marie Habedank, Johannes Meka, David Bierbach

**Affiliations:** ^1^Department of Biology and Ecology of Fishes, Leibniz Institute of Freshwater Ecology and Inland Fisheries, Berlin, Germany; ^2^Department of Evolution and Ecology, University of California, Davis, Davis, CA, United States; ^3^School of Life and Environmental Sciences A08, The University of Sydney, Sydney, NSW, Australia; ^4^Faculty of Life Sciences, Albrecht Daniel Thaer-Institute, Humboldt University of Berlin, Berlin, Germany; ^5^Cluster of Excellence “Science of Intelligence,” Technische Universität Berlin, Berlin, Germany

**Keywords:** developmental plasticity, swimming speed, thermal performance curve, *Poecilia formosa*, *Poecilia mexicana*, unisexual vertebrate

## Abstract

The capacity to compensate for environmental change determines population persistence and biogeography. In ectothermic organisms, performance at different temperatures can be strongly affected by temperatures experienced during early development. Such developmental plasticity is mediated through epigenetic mechanisms that induce phenotypic changes within the animal’s lifetime. However, epigenetic modifiers themselves are encoded by DNA so that developmental plasticity could itself be contingent on genetic diversity. In this study, we test the hypothesis that the capacity for developmental plasticity depends on a species’ among-individual genetic diversity. To test this, we exploited a unique species complex that contains both the clonal, genetically identical Amazon molly (*Poecilia formosa*), and the sexual, genetically diverse Atlantic molly (*Poecilia mexicana*). We predicted that the greater among-individual genetic diversity in the Atlantic molly may increase their capacity for developmental plasticity. We raised both clonal and sexual mollies at either warm (28°C) or cool (22°C) temperatures and then measured locomotor capacity (critical sustained swimming performance) and unforced movement in an open field across a temperature gradient that simulated environmental conditions often experienced by these species in the wild. In the clonal Amazon molly, differences in the developmental environment led to a shift in the thermal performance curve of unforced movement patterns, but much less so in maximal locomotor capacity. In contrast, the sexual Atlantic mollies exhibited the opposite pattern: developmental plasticity was present in maximal locomotor capacity, but not in unforced movement. Thus our data show that developmental plasticity in clones and their sexual, genetically more diverse sister species is trait dependent. This points toward mechanistic differences in how genetic diversity mediates plastic responses exhibited in different traits.

## Introduction

The early life environment can have pronounced and long-lasting effects on individual phenotypes (Atlasi and Stunnenberg, 2017; Hu and Barrett, 2017). Such developmental plasticity can allow an organism to better tailor their phenotypes for their future expected environments [“predictive adaptive hypothesis” (Bateson et al., 2014)] or better cope with rapid environmental perturbations later in life (Schulte et al., 2011). To understand the evolution of developmental plasticity, we need to understand when and how animals respond to early life environments and to what extent these responses allow animals to cope with later-in-life environmental conditions.

Organisms can respond to early life environments by adjusting their phenotype across numerous traits (Bozinovic et al., 2020). Reaction norms can be used to characterize the response of repeatedly expressed traits like behavioral or physiological traits across a range of environmental conditions (Sarkar and Fuller, 2003). The early developmental environment can lead to coordinated changes in whole suites of traits (Torres-Dowdall et al., 2012; Mateus et al., 2014); what is less clear is whether the plasticity underlying these traits is also matched. That is, will the reaction norm of one trait match the reaction norm of another in response to early environmental experiences? If some animals are more sensitive or better able to perceive environmental cues, then some authors have argued that plasticity should be consistent across different traits (Benus et al., 1987; Koolhaas et al., 1999; Whitman and Agrawal, 2009; Sih and Del Giudice, 2012; Forsman, 2015; Stamps and Biro, 2016). For example, in ectothermic animals, the thermal environment an animal experiences early in life can cause lifelong alterations to muscle contractile function resulting in coordinated effects on traits related to swimming capabilities (Hammill et al., 2004; Orczewska et al., 2010; Scott and Johnston, 2012; Le Roy et al., 2017). However, as different traits have different mechanistic bases it may instead be expected that there are differences in plasticity. Understanding whether and how patterns of plasticity are linked across different traits can therefore offer insight into the potential mechanistic underpinnings of these traits.

Epigenetic mechanisms are likely mediators of phenotypic changes such as developmental plasticity. For example, gene expression patterns can be altered by modifying access of transcriptional regulators to DNA (Whitfield et al., 2003; Aubin-Horth et al., 2005; Scott and Johnston, 2012; Ficz, 2015; Loughland et al., 2021), which can be mediated by changes in DNA-methylation patterns (Klose and Bird, 2006), histone binding (de Ruijter et al., 2003), or small RNA activity (Morris and Mattick, 2014). Even clonal, and hence genetically identical organisms often exhibit considerable phenotypic plasticity in response to variation in their environment (Doeringsfeld et al., 2004; Freund et al., 2013; Lynch and Kemp, 2014; Bierbach et al., 2017; Vogt, 2018). For example, the unisexual and genetically identical fish *Chrosomus eos-neogaeus* exhibited extensive variation in DNA methylation patterns across their genomes (Massicotte et al., 2011) that was correlated with environmental cues from their lake of origin (Massicotte and Angers, 2012). In the clonal Amazon molly (*Poecilia formosa*), several life history traits were strongly affected by salinity and temperature gradients experienced during developmental periods (Makowicz and Travis, 2020). Additionally, Amazon mollies raised in different social contexts developed different behavioral phenotypes (Bierbach et al., 2017). Developmental plasticity may therefore be especially relevant in such clonal organisms, as these animals do not have among-individual genetic variation to generate phenotypic variation.

However, even if phenotypic changes are mediated through epigenetic mechanisms, the shape of the reaction norm can also be altered in response to genotypic changes resulting from selection or genetic drift (Seebacher et al., 2012; Murren et al., 2014). Hence, variation in reaction norms can be mediated by variation in genetic and epigenetic mechanisms. For example, a single mutation determines whether *Manduca* caterpillars exhibit thermally sensitive pigmentation patterns (Suzuki and Nijhout, 2006) and if nematodes develop resource-sensitive variation in mouth morphologies (Bento et al., 2010). Additionally, the large number of proteins involved in the successful methylation (and demethylation) of DNA means that mutations at any number of nucleotides can alter the efficiency and/or specificity of this process (Klose and Bird, 2006; Campos et al., 2013). Natural selection or genetic drift may therefore influence the capacity for developmental plasticity in populations with greater among-individual genetic variation.

Here we test whether the capacity for developmental plasticity is linked across two phenotypic traits and whether plasticity depends on the presence of among-individual genetic variation. We raised closely related clonal and sexually reproducing fish species at two developmental temperatures to determine plasticity in thermal performance curves of swimming capacity and unforced movement. If functionally related traits are also mechanistically related, then we would predict that they would also show correlated patterns of developmental plasticity in response to early life environments. On the other hand, there is evidence for asymmetric thermal effects on thermal performance curves (Bozinovic et al., 2020), which instead predicts that patterns of plasticity are de-coupled. We investigated individual performance in two traits related to locomotion across a thermal gradient to compare the developmental plasticity: maximal swimming capacity measured as critical sustained swimming performance (U_crit_) and unforced movement in an open field. Both traits are relevant ecologically for these fish; U_crit_ reflects maximal physiological swimming capacity that could limit more extended movement like dispersal (Svendsen et al., 2017) or escape capabilities (Irschick et al., 2008). However, animals rarely move at maximum speed so that it is also relevant to determine temperature effects on the movement speed actually selected by individuals (Wilson et al., 2015). Both traits rely on muscle-powered locomotion, but differ in that U_crit_ is determined by the physiological capacities of the cardiovascular system, mitochondria and muscle, and unforced movement also reflects behavioral decisions that are under cognitive control (Stewart et al., 2013).

To investigate whether and how among-individual genetic diversity may alter patterns of developmental plasticity, we took advantage of a unique species complex that contains both clonal (genetically identical; the Amazon molly, *P. formosa*) and sexually reproducing (genetically diverse; Atlantic molly, *Poecilia mexicana*) fish (Laskowski et al., 2019). Amazon mollies are the first discovered clonal vertebrate (Hubbs and Hubbs, 1932; Schultz, 1973); they emerged from a single hybridization event between the Atlantic and sailfin molly (*Poecilia latipinna*) about 100,000 years ago and now reproduce gynogenetically (Lampert and Schartl, 2008; Stöck et al., 2010; Warren et al., 2018). The species requires sperm from one of their parental species (Atlantic or sailfin mollies) to stimulate embryonic development, but the paternal genetic material is not incorporated into the egg (but see Kallman, 1962; Rasch et al., 1965; Turner et al., 1980, for rare exceptions of male DNA fragment introgression). The offspring are therefore genetically identical to their mother and each other. The two species have essentially the same phylogenetic history, share half of their genome (Lampert and Schartl, 2008; Warren et al., 2018; Lu et al., 2021) and show a strong overlap in their ecological niche due to their sympatric occurrence as a result of the Amazon’s dependence on either sailfin or Atlantic mollies’ sperm for reproduction (Darnell and Abramoff, 1968; Schlupp et al., 2002; Scharnweber et al., 2011). This unique species complex thus allowed us to explore how the presence of among-individual genetic variation influences a species’ capacity for developmental plasticity in species that are otherwise ecologically identical. On one hand, sexual species such as the Atlantic molly harbor significantly more among-individual genetic variation (Warren et al., 2018; Lu et al., 2021) allowing natural selection to be more effective in shaping the capacity for developmental plasticity and so we predicted that we may see larger shifts in their reaction norms in response to early life environments. On the other hand, the lack of among-individual genetic variation in Amazon mollies leaves epigenetically induced phenotypic plasticity as the major avenue to adjust phenotypes to changing environmental conditions, and so an alternative prediction is that this clonal species would exhibit greater sensitivity, and hence plasticity, to early life environments. We are well aware of the limitations of two-species comparisons (Garland and Adolph, 1994), and we treat this comparison as exploratory and do not intend to infer adaptation.

## Materials and Methods

### Fish Breeding

**FIGURE 1:**
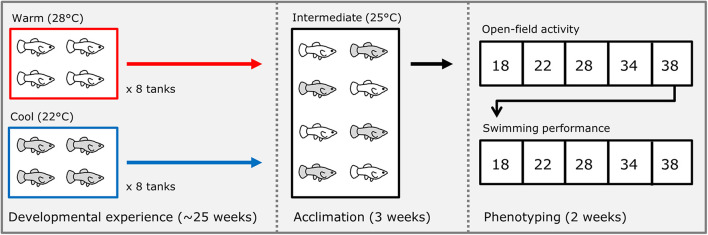
Schematic of experimental design. Immediately after birth, broods of *Poecilia formosa* and *Poecilia mexicana* were split into either a “warm” or “cool” treatment where they developed for approximately 25 weeks. After this time, fish were then acclimated to an intermediate temperature for 3 weeks. After acclimation, each fish was tested for their open field movement and swimming performance at five different temperatures (in random order) over the course of 2 weeks.

We isolated several individual pregnant females of each species (housed at 25°C through their lifetime), and immediately after they gave birth, we split broods into groups of 4 sibs each; half of these groups were placed into a warm (28°C) treatment and half into a cool (22°C) treatment (Figure 1). Each group was maintained in a 38-liter aquarium with gravel and a plastic plant for shelter. These treatments were chosen as ecologically relevant temperatures the fish would experience seasonally in the wild (Schlupp et al., 2002; Costa and Schlupp, 2010). We generated a total of 16 groups of 4 sibs of the Amazon mollies (*P. formosa*, 8 groups per treatment) from 4 different broods (mothers that were sisters), and 18 groups (9 groups per treatment) of 4 sibs each for the Atlantic mollies (*P. mexicana*) from 5 different broods (mothers). Each treatment group was reared at their respective developmental thermal environment treatments until they were 25–27 weeks old. Fish were then acclimated to a common-garden, intermediate temperature (25°C) for 3 weeks. We conducted the common-garden acclimation treatment to reduce the effects of short-term reversible acclimation from that of the long-term effects of the developmental treatment that we were interested in here. Individuals were acclimated in individual clear plastic bottles (10 cm diameter) placed within a communal tank. The bottles had holes in their sides that were small enough to prevent fish from passing through while still allowing visual and chemical cues to pass among individuals. This set-up allowed us to follow individuals without the need to invasively mark them before phenotyping trials, and it limited handling stress because fish did not need to be netted for each trial (see below). After 3 weeks in acclimation, we measured unforced movement in an open field and locomotor capacity (see below) in each individual.

Experimental fish of both species were lab-reared descendants of wild-caught fish bred in the laboratory for several generations. Founding individuals of both species were originally collected near the Mexican city of Tampico, where both species occur in sympatry. Regular molecular checks confirmed that all *P. formosa* individuals are clones (M. Schartl, personal communication), and the *P. mexicana* populations have been regularly supplied with new individuals from the wild to maintain levels of natural standing genetic variation; however, this procedure was stopped at least five generations ago to minimize uncontrollable cross-generational epigenetic effects (Kelley et al., 2021) brought in by differences in individual origin.

Throughout the experiment fish were fed twice daily on flake food (TetraMin, tropical fish flake food) and maintained on 12:12 L:D light cycle. On trial days, fish were not fed until after trials were completed. Measurements were staggered over the course of several weeks to ensure that individuals born at different times were of the same age at the time of measurement. We only included data from females of the Atlantic mollies (50 out of 69 experimental animals) as the Amazon molly is an all-female species. Additionally, males are generally considerably smaller and have a different body shape compared to females which could influence their swimming behaviors. In total we collected phenotypic data from 50 Atlantic mollies and 59 Amazon mollies. All behavioral protocols complied with German law and were approved by the Berlin Landesamt für Gesundheit und Soziales (GO 124/14).

### Unforced Movement in an Open Field

Behavioral traits, such as unforced movement, are arguably some of the most plastic phenotypes an animal can exhibit and so might be especially sensitive to early environmental cues. Alternatively, because behavior is the result of many sensory, neural and cognitive inputs it may respond most strongly to the immediate environment and may not exhibit long-lasting shifts in response to early life environments. We measured unforced movement of each individual in an open field (white circular arena 48.5 cm diameter, water level 6 cm) (Bierbach et al., 2017) at 18, 22, 28, 34, and 38°C acute test temperatures. We chose these temperatures as they cover the range of temperatures that both species could encounter in the wild (Schlupp et al., 2002; Costa and Schlupp, 2010) and are well within the physiological tolerated range of each species (Bierbach et al., 2010). Fish were measured once per day for 5 days, each day at a different acute test temperature. The order of the temperatures was randomly assigned. In between trials, fish were returned to their individual bottles at the acclimation temperature. Before experiments, we familiarized each individual with the open field arena by conducting a single assay at 25°C to avoid confounding our measure of movement with an effect of novelty to the unfamiliar environment. We did not include data from these pre-trials in the analysis.

To perform an open field trial, an individual bottle containing a fish was removed from the acclimation tank and gently poured into a dark plastic cylinder at the center of the arena. The fish was allowed to rest for 1 min, after which time we gently lifted the cylinder and recorded the behavior of the fish for the next 5 min using a webcam (C920, Logitech, United States). After 5 min, we removed the fish and placed it back in its bottle in the acclimation tank. The water in the arena was replaced in between each trial to minimize chemical cues and to maintain the appropriate temperature by replacing water from a sump tank at the appropriate temperature for the day. Fish were tested in random order. The videos of the open field movement were analyzed using EthoVision 11TX software (Noldus Information Technologies, Inc., Netherlands) from which we extracted the mean velocity of the animal (in body lengths s^–1^) over the 5-min trial as our measure of movement. Note that other measures such as total distance swam during the trial yielded essentially identical results; see Supplementary Table 1. After the trials, we took a digital photograph of each fish, from which we measured the standard length of each fish to the nearest mm.

### Swimming Performance

Maximum locomotor capacity is a whole-animal performance trait that is determined to a large extent by muscle contractile function and there is evidence from multiple fish species (Hammill et al., 2004; Seebacher et al., 2012), including the closely related guppy, *P. reticulata* (Le Roy et al., 2017) that it responds plastically to early life environments. In the week following the open field tests, we measured maximal locomotor capacity as the critical sustained swimming speed (*U*_crit_) (Kolok, 1999) of each individual at the same acute test temperatures (18, 22, 28, 34, and 38°C) in random order. We measured locomotor capacity because it integrates several underlying physiological systems, and it is closely related to fitness by increasing success in predator escape, prey capture, and increasing reproductive success (Irschick et al., 2008). *U*_crit_ was measured according to published protocols (Seebacher et al., 2015) in a Blazka-style swimming flume consisting of a cylindrical clear Perspex flume (150 mm length and 38 mm diameter). The flume was fitted tightly over the intake end of a submersible pump (12V DC, iL500, Rule, Hertfordshire, United Kingdom). A bundle of hollow straws at the inlet end of flume helped maintain laminar flow. The flume and pump were submerged in a plastic tank (38 cm by 62 cm) that contained water with the appropriate temperature for each trial. We controlled water flow speed by changing the voltage input into the pump with a variable DC power source (NP9615; Manson Engineering Industrial, Hong Kong, SAR China). The water flow in each flume was measured in real-time by a flow meter (DigiFlow 6710 M, Savant Electronics, Taichung, Taiwan) connected to the outlet of each pump. Fish swam at an initial flow rate of 0.06 m s^–1^ for 20 min followed by an increase in flow speed by 0.02 m s^–1^ every 5 min until the fish could no longer hold their position in the water column. When fish fell back onto the grid, the flow was stopped for 5–10 s before restarting and increasing the speed to the previous setting again. We terminated the trial when fish stopped swimming for the second time. Fish were rested for at least 24 h between swimming trials. We report U_crit_ as body length per second (BL s^–1^).

### Statistical Analysis

We tested for shifts in the reaction norms of locomotor capacity and movement due to the developmental thermal environment using linear mixed models. We first ran one model for each trait (locomotor capacity and movement in an open field) to test for an overall three-way interaction between species (Atlantic or Amazon), developmental temperature (22 or 28°C) and acute test temperature (18, 22, 28, 34, and 38°C). To determine whether species differed, we tested for the three-way interaction (“species × developmental temperature × test temperature” and “species × developmental temperature × test temperature^2^”) of both linear and quadratic effects of test temperature; the quadratic effect captures the curvature in the performance curve of the traits, and the linear term indicates the slope of the reaction norm of the trait. We additionally included the fixed effects of observation order (day 1 – 5) and body length. Individual fish ID and Mother ID were included as random effects to account for the multiple observations per fish and brood.

After testing for the three-way interactions, we investigated differences in developmental plasticity within each species and each trait separately. In each model we included the effects of developmental temperature, linear and quadratic effects of test temperature, interactions between these and developmental temperature, and the effects of observation order and body length. Individual fish ID and Mother ID were included as random effects. In preliminary analyses we tested different random structures including random intercepts for each fish, tank (group of four siblings) and mother, random slopes and intercepts for each fish, and random curves, slopes and intercepts for each fish. However, in all cases the best random structure only contained random intercepts for each individual fish and mother, which we therefore used for all models (see Supplementary Tables 2,3). We additionally estimated both a marginal *R*^2^ (proportion of total variance explained by the fixed effects) and conditional *R*^2^ (proportion of total variance explained by the fixed and random effects) value for each model according to Nakagawa and Schielzeth (2017). We did not remove non-significant terms from our full models as we were interested *a priori* in all effects. We centered and scaled to unit variance the continuous variables (mean velocity, *U*_crit_, and body length) before analysis to enable comparisons of effect estimates (Schielzeth, 2010). Acute test temperature was centered but not scaled to make the intercept more interpretable (Schielzeth, 2010). Inspection of the residuals confirmed that our models met the assumptions of a Gaussian error distribution with homogeneous variance. The significance of fixed and random effects was assessed using the log-likelihood ratio of a model that contained the effect of interest to a model that did not. Where interactions (e.g., between developmental temperature and test temperature) were significant, we did not test for the significance of the main effects (e.g., developmental temperature) as this would require removing the significant two-way interaction from the model. Models were run using the lme4 package in R (Bates et al., 2015).

To get an overall measure of each individual’s swimming performance across all test temperatures, we analyzed thermal performance curves of *U*_crit_ by fitting quadratic equations to the data from each fish (Seebacher et al., 2015), and then setting the first differential to zero to obtain the mode of the curve (i.e., the temperature at which maximal *U*_crit_ occurred). We obtained the performance breadth (i.e., the temperature range over which *U*_crit_ was >80% of maximal) by reducing the maximum of the fitted curve for each fish to 80% and then calculating quadratic roots (Seebacher et al., 2015). We were unable to fit curves for movement in an open field as this behavior did not follow a typical quadratic curve shape (see section “Results”).

Finally, to test whether and how the two traits were related to each other, we estimated among- and within-individual correlations between the two traits for each species separately. We used multivariate mixed models with *U*_crit_ and mean velocity as the response variables and individual included as a random effect. We attempted to include mother as an additional random effect, however, these models failed to converge likely due to the relatively small variance attributable to mother (see section “Results”) and so was removed. Each trait was centered and scaled to unit variance prior to analysis so the resulting covariance estimates are equivalent to correlation coefficients (Dingemanse and Dochtermann, 2013). We used the MCMCglmm package in R (Hadfield, 2010) and ran chains of 400,000 iterations with a burn-in of 1,000 and thinning every 200 samples. We assumed Gaussian error distributions for each trait and used parameter-expanded priors and preliminary analyses indicated our results were not sensitive to prior specification. Inspection of the posterior plots of five independent chains indicated our models achieved good mixing. We interpreted a correlation coefficient as significantly different from zero if the resulting 95% credible interval did not overlap zero. Data and R code used to generate the results are provided as Supplementary material.

## Results

### Maximal Locomotor Capacity

**FIGURE 2:**
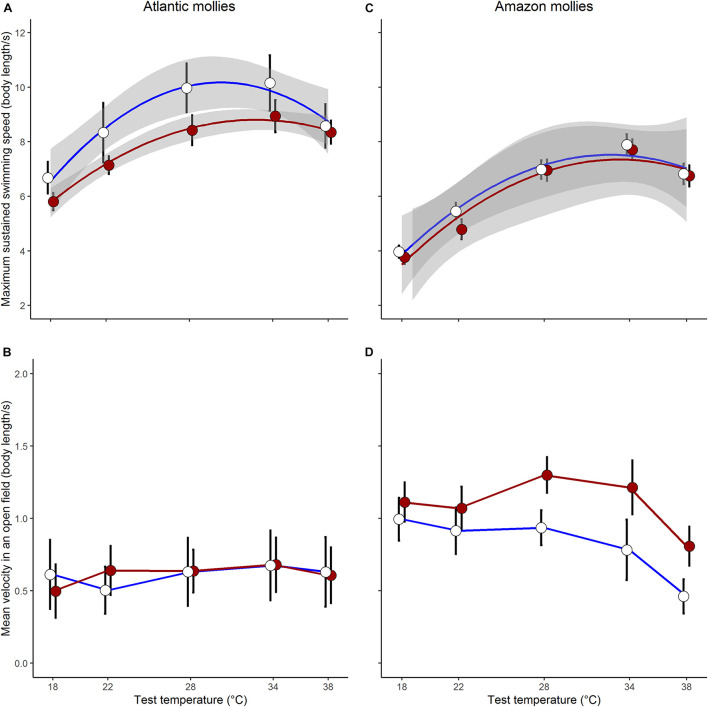
Plasticity in locomotor traits. Sexually reproducing Atlantic mollies exhibited developmental plasticity in swimming performance **(A)** but not open field movement **(B)** in response to different developmental thermal environments. Clonal amazon mollies showed no developmental plasticity in swimming performance **(C)** but did in open field movement **(D)**. Shown are means ± SE; *N* = 26, 24 for cold, hot Atlantic mollies, respectively; and *N* = 28, 31 for cold, hot Amazon mollies, respectively. The gray bands in panel **(A,C)** indicate the 95% CI from the fitted quadratic curve; we were unable to fit similar curves to the movement data as it did not follow a typical quadratic form.

As predicted, there was an indication that the two species differed in their capacity for developmental plasticity in response to early life thermal environment (Dev.temp × Species × Test.temp^2^: log-likelihood ratio (LLR) = 3.65, *p* = 0.055; see full results of the three-way interaction model in Supplementary Table 4). In general, the sexually reproducing Atlantic molly exhibited a greater capacity for developmental plasticity in *U*_crit_ compared to the clonal Amazon molly (Figures 2A,C).

**FIGURE 3:**
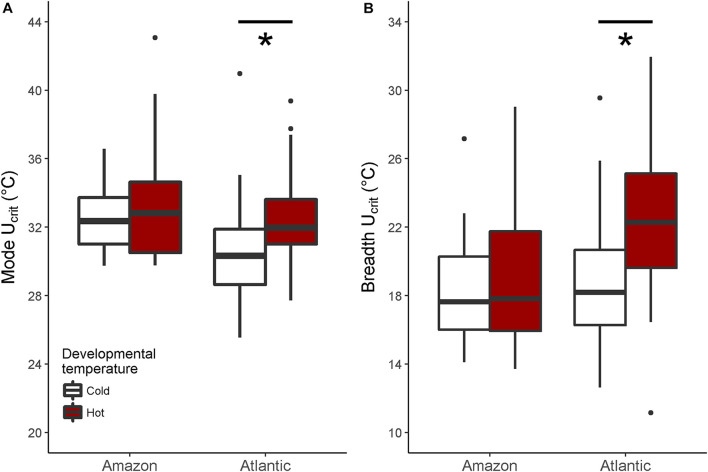
Mode and breath of the thermal performance curves. In Atlantic mollies, different developmental temperatures shifted the mode (i.e., the temperature at which maximal swimming performance occurred) significantly **(A)** and increased the performance breadth (i.e., 80% maximal performance) **(B)**. Shown are boxplots where the box indicates the upper and lower quartiles, the dark middle line indicates the median, the whiskers indicate 1.5 times the inter-quartile range and asterixes denote significant differences. There were no significant effects of developmental temperature on performance curve mode or breadth in clonal Amazon mollies. Any values outside that range are shown as points. *N* = 26, 24 for cold, hot Atlantic mollies, respectively; and *N* = 28, 31 for cold, hot Amazon mollies respectively.

When investigating *U*_crit_ within each species separately, we found that the curvature of the swimming thermal performance curve in sexual Atlantic mollies depended on the developmental temperature (Dev.temp × Test.temp^2^ interaction, Table 1) indicating that early experience altered the thermal sensitivity of locomotor capacity later in life (Figure 2A). The combined fixed effects in our model explained nearly half of the total variation in *U*_crit_ (marginal *R*^2^ = 0.43, Table 1), although there was still considerable variation among individuals and families (mothers) that explained an additional 31% of the total variance (conditional *R*^2^ – marginal *R*^2^, Table 1). As predicted, Atlantic mollies raised at the higher developmental temperature achieved peak performance at a higher temperature (mode, Figure 3A) and maintained performance across a broader range of temperatures (breadth, Figure 3B) compared to fish raised at the cooler developmental temperature (mode: *t* = 3.13, *p* = 0.003; breadth: *t* = 3.40, *p* = 0.001). However, maximum performance was overall lower in fish from the high developmental temperatures compared to those from the low treatment (Figure 2A and Table 1).

**TABLE 1:** Linear mixed effect model predicting mean velocity and critical sustained swimming speed (*U*_crit_) in the **Atlantic mollies**.

**Effect**	**Estimate (± s.e.)**	***t*−value**	**LLR**	***p*−value**
**Critical sustained swimming speed in a flume (marginal *R*^2^ = 0.41; conditional *R*^2^ = 0.72)^a^**				
Intercept	1.26 (0.20)	6.27		
**Length**	**−0.17 (0.07)**	**−2.45**	**6.00**	**0.014**
Observation	−0.008 (0.03)	–0.31	0.15	0.70
Dev.temp (warm)	−0.67 (0.20)	–3.37		
**Test.temp**	**0.05 (0.007)**	**7.42**	**101.07**	**< 0.001**
Test.temp^2^	−0.01 (0.001)	–9.13		
Dev.temp × Test.temp	0.01 (0.009)	1.13	1.32	0.25
**Dev.temp × Test.temp^2^**	**0.005 (0.002)**	**2.66**	**7.12**	**0.007**
Individual variance	0.212			
Mother variance	0.100			
Residual variance	0.293			
Adjusted repeatability^b^	0.35			
**Mean velocity in an open field (marginal *R*^2^ = 0.12, conditional *R*^2^ = 0.55)^a^**				
Intercept	−0.61 (0.20)	–2.99		
**Length**	**−0.28 (0.09)**	**−3.14**	**9.59**	**0.002**
Observation	−0.05 (0.03)	–1.71	3.17	0.07
Dev.temp (warm)	0.35 (0.25)	1.39	1.22	0.27
**Test.temp**	**0.01 (0.008)**	**1.35**	**3.76**	**0.05**
Test.temp^2^	<0.001 (0.001)	0.11	0.66	0.42
Dev.temp × Test.temp	<0.001 (0.01)	0.07	0.004	0.94
Dev.temp × Test.temp^2^	−0.002 (0.002)	–0.99	1.01	0.31
Individual intercepts variance	0.367			
Mother variance	0.053			
Residual	0.446			
Adjusted repeatability^b^	0.42			

*Responses and length were centered and scaled to unit variance, and test temperature was centered prior to analysis. Significance of effects was estimated using a log-likelihood ratio test on nested models; in models where a two-way interaction was significant, we did not test the significance of an involved main effect (see section “Materials and Methods” for more details). Estimates significant at the *p* < 0.05 level are bolded.*

*^a^Marginal *R*^2^ describes the proportion of the total variance that is explained by the fixed effects in the model whereas conditional *R*^2^ describes the proportion of total variance that is explained by the combined fixed and random effects in the model.*

*^b^Repeatability was estimated as the proportion of the remaining variance (not explained by the fixed effects) that was attributable to differences in individual intercepts.*

In comparison, the *U*_crit_ of the clonal Amazon mollies was not significantly affected by their developmental thermal environment. Neither the linear nor the quadratic effects of test temperature interacted with developmental temperature (Table 2 and Figure 2C). There was an overall effect of developmental temperature on *U*_crit_ (Table 2), but this effect was small biologically (Figure 2C). Also, there was no difference in *U*_crit_ mode (*t* = 0.65, *p* = 0.52) or breadth (*t* = 0.98, *p* = 0.33) between the different developmental temperatures (Figure 3). Our model explained a large portion of the total variance in swimming performance (marginal *R*^2^ = 0.76), and the additional portion of variance explained by individual identity was low (4%; conditional *R*^2^ – marginal *R*^2^, Table 2).

**TABLE 2:** Linear mixed effect model predicting mean velocity and critical sustained swimming speed (*U*_crit_) in the **Amazon mollies**.

**Effect**	**Estimate (± s.e.)**	***t*−value**	**LLR**	***p*−value**
**Critical sustained swimming speed in a flume (marginal *R*^2^ = 0.76, conditional *R*^2^ = 0.80)^a^**				
Intercept	0.07 (0.06)	1.10		
**Length**	**−0.34 (0.05)**	**−6.93**	**36.81**	**< 0.001**
**Observation**	**0.06 (0.02)**	**3.45**	**11.79**	**< 0.001**
**Dev.temp (warm)**	**−0.14 (0.08)**	**−1.79**	**4.35**	**0.04**
**Test.temp**	**0.07 (0.004)**	**17.73**	**345.92**	**< 0.001**
**Test.temp^2^**	**−0.007 (<0.001)**	**−8.47**	**96.94**	**< 0.001**
Dev.temp × Test.temp	0.007 (0.006)	1.30	1.73	0.18
Dev.temp × Test.temp^2^	<0.001 (0.001)	0.44	0.19	0.65
Individual variance	0.020			
Mother variance	0.001			
Residual variance	0.119			
Adjusted repeatability^b^	0.14			
**Mean velocity in an open field (marginal *R*^2^ = 0.30; conditional *R*^2^ = 0.56)^a^**				
Intercept	0.38 (0.13)	2.84		
**Length**	**−0.44 (0.13)**	**−3.47**	**11.58**	**< 0.001**
Observation	−0.04 (0.03)	–1.42	2.07	0.35
Dev.temp (warm)	0.68 (0.03)	4.02		
Test.temp	−0.05 (0.007)	–6.71		
**Test.temp^2^**	**−0.004 (0.001)**	**−3.12**	**32.39**	**< 0.001**
**Dev.temp** × **Test.temp**	**0.03 (0.01)**	**3.08**	**9.45**	**0.002**
Dev.temp × Test.temp^2^	−0.002 (0.002)	–1.31	1.76	0.18
Individual variance	0.207			
Mother variance	0.007			
Residual variance	0.358			
Adjusted repeatability^b^	0.36			

*Responses and length were centered and scaled to unit variance, and test temperature was centered prior to analysis. Significance of effects was estimated using a log-likelihood ratio test on nested models; in models where a two-way interaction was significant, we did not test the significance of an involved main effect (see section “Materials and Methods” for more details). Estimates significant at the *p* < 0.05 level are bolded.*

*^a^Marginal *R*^2^ describes the proportion of the total variance that is explained by the fixed effects in the model whereas conditional *R*^2^ describes the proportion of total variance that is explained by the combined fixed and random effects in the model.*

*^b^Repeatability was estimated as the proportion of the remaining variance (not explained by the fixed effects) that was attributable to differences in individual intercepts.*

### Movement

The effect of different developmental temperatures on unforced movement in an open field differed between the two species (Dev.temp × Species × Test.temp LLR = 4.24, *p* = 0.039, Supplementary Table 5). Interestingly, however, we found the opposite pattern to *U*_crit_ in the plasticity of movement in an open field: the clonal Amazon molly exhibited greater plasticity in response to the developmental environment compared to the sexual Atlantic molly. Movement of clonal Amazon mollies depended on the developmental temperature: fish raised in warmer environments exhibited greater movement at warmer temperatures compared to fish raised at cooler temperatures (Dev.temp × Test.temp interaction; Table 2 and Figure 2D). The fixed effects in our model explained a lower proportion of the total variance (marginal *R*^2^ = 0.30, Table 2) compared to that in the analysis of *U*_crit_, although individual identity explained a much larger portion of variation in movement (26%, conditional *R*^2^ – marginal *R*^2^) compared to *U*_crit_.

In contrast, the sexual Atlantic molly showed low levels of movement in an open field, and movement was only marginally affected by test temperature (Figure 2B and Table 1). There was no effect of developmental temperature. The fixed effects in our model explained only 12% of the total variation in movement (marginal *R*^2^, Table 1), although there was considerable variation among individuals (43% of the total variation; conditional *R*^2^ – marginal *R*^2^, Table 1).

*U*_crit_ and movement were weakly correlated at the among-individual level showing that Atlantic mollies that had higher locomotor capacity on average also were more active in the open field (*R* = 0.25, 95% CI: [0.09, 0.44]). There was no relationship at the within-individual level (*R* = 0.02 [−0.06, 0.10]). In the Amazon mollies, there was no evidence that these two traits were correlated at either the among- (*R* = 0.03 [−0.01, 0.11]) or within-individual level (*R* = −0.03 [−0.12, 0.07]).

## Discussion

Here we show that the capacity for developmental plasticity differs between two related species and across two related traits within a species. We found clear evidence that the sexually reproducing Atlantic molly exhibited shifts in the reaction norm of their physiological swimming performance, but not of their movement levels. In contrast, the clonal Amazon molly exhibited the exact opposite pattern where their unforced movement was more plastic than their swimming performance. Therefore, we show that higher levels of among-individual genetic variation as seen in Atlantic mollies only leads to more pronounced developmental plasticity compared to the genetically identical Amazon mollies in a very trait specific manner even after fish had experienced long periods of very different thermal regimes (22 vs. 28°C for 25 weeks prior to testing). Furthermore, our data indicate that the mechanistic basis underlying these two traits might have different susceptibilities to epigenetic modifications and that there is possibly an interaction between genetic variation and epigenetic mechanisms, be that as a result of genetic diversity among individuals or genetic differences between the species.

Maximal locomotor capacity in the sexually reproducing Atlantic molly exhibited developmental plasticity, shifting location (mode) and shape (breadth) of their performance curves. This developmentally induced shift in swimming thermal performance curves meant that there was no difference in performance when developmental temperatures coincided with acute temperatures, that is, the 22°C developed fish measured at 22°C performed as well as 28°C developed fish measured at 28°C. Hence, developmental plasticity equalized performance so that it stayed constant at the anticipated environmental conditions later in life (Kawecki, 2000). Similar canalization occurred in guppies (*P. reticulata*), but only after two generations (Le Roy et al., 2017), and it may protect populations from environmental perturbations rather than matching phenotypes to prevalent environmental conditions as predicted by the “predictive adaptive hypothesis” (Bateson et al., 2014; Le Roy et al., 2017). Even though there were shifts in the performance curves in response to the early life thermal environment in the sexually reproducing fish, the maximum of each performance curve did not occur at the acute temperature that matched the developmental temperature as predicted by the “predictive adaptive hypothesis.” Rather, both warm and cold reared fish achieved their greatest performance at temperatures warmer than their developmental temperatures.

In contrast, there was no shift in maximum locomotor performance in clonal Amazon mollies reared at different temperatures. We exposed fish to their respective developmental conditions over a relatively long period that would have included the developmental stages that are most sensitive to external temperature signals (Campos et al., 2012). Hence, lack of developmental plasticity is unlikely to be an artifact of the experimental treatment. The difference in the patterns of developmental plasticity may be due to genetic differences between the species even though the species share a large part of their genomes. Half of the Amazon molly’s genome is from its Atlantic molly ancestor; the other half is from its sailfin molly ancestor (Lampert and Schartl, 2008; Stöck et al., 2010; Warren et al., 2018; Lu et al., 2021). Additionally, Amazon mollies require sperm from one of its parental species (Atlantic and sailfin molly), and Amazon and Atlantic mollies are sympatric for much of their ranges (Schlupp et al., 2002). The co-existence of clonal and sexually reproducing lineages is interesting, and it may be that partitioning of ecological niches facilitates this co-existence. However, there do not appear to be differences in their competitive abilities (da Barbiano et al., 2010, 2013; Scharnweber et al., 2011) or parasite loads (Tobler and Schlupp, 2005; Tobler et al., 2005). Hence, the major difference between these two species is that half of their genomes differ, and that they differ in among-individual genetic diversity. It is likely, therefore, that an interaction between genetic - at the (half)species and/or among-individual levels - and epigenetic mechanisms caused the differences in developmental plasticity in Amazon and Atlantic mollies.

Genetic and epigenetic mechanisms can interact at multiple levels (Ashe et al., 2021). Importantly, epigenetic mechanisms are themselves not independent from genetics, because epigenetic modifiers such as DNA methyltransferases and histone deacetylases are themselves encoded by DNA (Campos et al., 2012). Additionally, higher recombination rates are related to higher GC content (Stapley et al., 2017), which implies that a sexually reproducing species (with higher recombination), such as the Atlantic molly may have increased susceptibility to DNA methylation due to their increased GC content (Gelfman et al., 2013) compared to the clonal, non-recombining Amazon molly. Therefore, one possible explanation for the difference in the patterns of developmental plasticity in locomotor capacity in these two species is that developmental plasticity and canalization can be modulated by genetic diversity. The epigenome is now viewed as having at least as important an influence in shaping phenotypes as the DNA nucleotide sequence (Forsman, 2015; Ashe et al., 2021). Epigenetic modifications in response to different early life environments may not be independent from genetic diversity, and genetically mediated diversity in the molecular machinery that confers epigenetic changes (Taudt et al., 2016) could increase the efficacy of developmental plasticity at least for some traits. Epigenetic processes may also be linked to genetics and selection because epigenetic states can be heritable, and the resulting plasticity and phenotypic variance can affect selection (Stajic and Jansen, 2021). Our data indicate that there are interactions between genetic and epigenetic mechanisms in determining developmental plasticity, but their exact manifestation and consequences must await further experimentation.

In addition to the difference in among-individual genetic diversity between Atlantic and Amazon mollies, there are other genetic factors that could play a role. In particular, while there was no genetic diversity among Amazon molly individuals within the same clonal lineage, there is high genetic diversity at the within-individual level (Lampert and Schartl, 2008; da Barbiano et al., 2013; Warren et al., 2018). The Amazon molly is a “frozen hybrid” that originated from a single hybridization event between a female Atlantic and a male sailfin molly and so exhibits extremely high heterozygosity, which is greater even than in either of its two parental species (Warren et al., 2018). This heterozygosity is one potential explanation for why clonal fish have persisted so long despite their inability to generate new genetic variation through sexual recombination. It is important to note, however, that the genome of the Amazon molly shows similar patterns of gene conversion, mutation accumulation and transposable element activity as genomes of both the Atlantic and sailfin molly (Warren et al., 2018). In addition to the accumulation of mutations, the rare introgression of paternal DNA can also generate diversity among clonal lineages (Schartl et al., 1995; Nanda et al., 2007; Warren et al., 2018). Genetic diversification between lineages generates clonal sorting, whereby the most fit clonal lineages are more likely to persist in a given environment (Vrijenhoek, 1979; Dawley and Bogart, 1989). These patterns of genetic diversification raise the possibility that differences in developmental plasticity between Atlantic and Amazon mollies are not just a by-product of differences in genetic diversity, but may have emerged as a result of selection or genetic drift (see Makowicz and Travis, 2020; Lu et al., 2021). To resolve these questions, it would be informative to also examine patterns of developmental plasticity in the second ancestral species, the sailfin molly (*Poecilia latipinna*), and in populations of the Amazon molly sampled across their geographic range.

We found no evidence for developmental plasticity in movement levels in the Atlantic molly demonstrating that the greater among-individual genetic diversity in this species is not the only contributor to developmental plasticity. As all fish will automatically swim when placed in running water, maximal locomotor capacity is principally constrained by intrinsic muscle function mediated by muscle fiber type expression and calcium cycling, for example (Josephson, 1993; Gordon et al., 2000; Seebacher and Walter, 2012), which are known to be modified epigenetically (McGee and Hargreaves, 2011; Campos et al., 2013; Simmonds and Seebacher, 2017). In contrast, behaviors such as unforced movement are likely modulated by a broader range of physiological systems including neuroendocrine and sensory inputs, nutritional state, metabolic rates, in addition to muscle function (Akre and Johnsen, 2014). Considering the different mechanisms underlying maximal locomotor capacity and movement in an open field, it is not surprising that these traits are only weakly correlated with one another at the among-individual level and not at all at the within-individual level. The two traits might respond differently to developmental inputs and other environmental cues might play a more important role in influencing this behavior in the Atlantic mollies (Forsman, 2015). For example, it is possible that the Atlantic mollies may have perceived the open field as riskier than the Amazon mollies did, and therefore maintained low movement levels regardless of temperature. We attempted to limit this possibility by giving the fish exposure to the arena the day before testing, and we would expect there to be evidence of some habituation effect as the fish became more familiar with the (initially) novel environment; however, there was no clear effect of repeated testing on movement levels. The complexity of behavioral traits is further underscored by the fact that individuals of both species consistently differed in their movement. This pattern of consistent behavioral differences was similar to previous findings showing differences in behavior among genetically identical Amazon molly individuals reared under identical environmental conditions (Bierbach et al., 2017).

In conclusion, we demonstrated that two seemingly related traits in two closely related species exhibit very different patterns of developmental plasticity. Our results suggest that greater (among-individual) genetic variation may enhance the capacity for developmental plasticity in a physiological trait but may not be necessary for plasticity in behavioral traits. These results have important implications on how animals respond to rapid environmental change, and how populations that face different environments may diverge genetically *via* genetic assimilation of epigenetically acquired characters.

## Data Availability Statement

The original contributions presented in the study are included in the article/Supplementary Material, further inquiries can be directed to the corresponding author/s.

## Ethics Statement

The animal study was reviewed and approved by the Berlin Landesamt für Gesundheit und Soziales (GO 124/14).

## Author Contributions

KL, FS, and DB developed the study and analyzed the data. DB, MH, and JM performed the experiments, KL, FS, and DB wrote the manuscript with input from all authors. All authors contributed to the article and approved the submitted version.

## Conflict of Interest

The authors declare that the research was conducted in the absence of any commercial or financial relationships that could be construed as a potential conflict of interest.

## Publisher’s Note

All claims expressed in this article are solely those of the authors and do not necessarily represent those of their affiliated organizations, or those of the publisher, the editors and the reviewers. Any product that may be evaluated in this article, or claim that may be made by its manufacturer, is not guaranteed or endorsed by the publisher.
