# Supplementary material for: Two Locomotor Traits Show Different Patterns of Developmental Plasticity Between Closely Related Clonal and Sexual Fish
Source: Front Physiol. 2021 Oct 12;12:740604. doi: 10.3389/fphys.2021.740604 (PMC8546259; doi:10.3389/fphys.2021.740604)
Supplement: Supplementary file 1 [file Table_1.DOCX]

Supplementary Material

# Supplementary Tables

**Supplemental Table 1.** Results of analysis using total distance swam in the open field arena (as opposed to mean velocity) for the Atlantic and Amazon mollies.

| Effect | Estimate (± s.e.) | d.f. | t-value | p-value |
| --- | --- | --- | --- | --- |
| *Atlantic Mollies* | | | | |
| Intercept | -0.14 (00.21) | 13.22 | -0.66 |  |
| Length | -0.16 (0.12) | 46.26 | -1.42 | 0.16 |
| Observation | -0.08 (0.03) | 195.00 | -2.36 | 0.02 |
| Dev.temp (warm) | 0.35 (0.26) | 76.54 | 1.35 | 0.18 |
| Test.temp | 0.01 (0.008) | 195.00 | 1.58 | 0.11 |
| Test.temp^2^ | -0.0001 (0.001) | 195.00 | -0.08 | 0.93 |
| Dev.temp x Test.temp | -0.0009 (0.01) | 195.00 | -0.08 | 0.93 |
| Dev.temp x Test.temp^2^ | -0.002 (0.002) | 195.00 | -1.05 | 0.29 |
| Individual variance | 0.398 |  |  |  |
| Mother variance | 0.071 |  |  |  |
| Residual variance | 0.512 |  |  |  |
| *Amazon mollies* | | | | |
| Intercept | -0.04 (0.15) | 21.29 | -0.28 |  |
| Length | -0.14 (0.08) | 54.78 | -1.78 | 0.08 |
| Observation | -0.02 (0.03) | 222.53 | -0.54 | 0.58 |
| Dev.temp (warm) | 0.78 (0.19) | 113.63 | 4.04 | <0.001 |
| Test.temp | -0.05 (0.008) | 222.97 | -6.64 | <0.001 |
| Test.temp^2^ | -0.005 (0.001) | 222.62 | -3.22 | 0.001 |
| Dev.temp x Test.temp | 0.03 (0.01) | 222.86 | 3.01 | 0.002 |
| Dev.temp x Test.temp^2^ | -0.003 (0.002) | 222.61 | -1.40 | 0.16 |
| Individual intercepts variance | 0.266 |  |  |  |
| Mother variance | 0.008 |  |  |  |
| Residual | 0.479 |  |  |  |

**Supplemental Table 2.** Different random effects structure for models predicting mean velocity and maximum sustained swimming speed in the **Atlantic mollies**. All models contained the same fixed effect structure (~ Dev.temp*Test.temp + Dev.temp*Test.temp^2^ + Length + Obs.order). Mean velocity, swimming speed and length were centered and scaled to unit variance. Test temperature was centered (but not scaled). For reporting, we chose models that only contained the random effect for Individual fish and Mother identity as these factors were inherent to the design and there was not strong support that including individual/maternal variation in linear slopes or curves (across Test.temp) improved model fit. ‘Ref M’ refers to the reference model (M) against which the model was compared. ‘M df’ are the number of degrees of freedom in the model, LLR refers to the log-likelihood ratio between the model and the reference model.

| M | Random structure | Ref M | M d.f. | AIC | LLR | p-value |
| --- | --- | --- | --- | --- | --- | --- |
| *Mean velocity in an open field (BL/s)* | | | | | | |
| 0 | -- | -- | 8 | 738.25 |  |  |
| 1 | (1\|ID) | 0 | 9 | 665.72 | 74.53 | <0.0001 |
| **2** | **(1\|Mother/ID)** | **1** | **10** | **665.54** | **2.21** | **0.04** |
| 3 | (1 + Test.temp\|ID) | 1 | 10 | 666.00 | 1.715 | 0.09 |
| 4 | (1 + (Test.temp + Test.temp^2^)\|ID) | 3 | 11 | 667.71 | 0.289 | 0.28 |
| *Maximum sustained swimming speed in a flume (BL/s)* | | | | | | |
| 0 | -- | -- | 8 | 626.54 |  |  |
| 1 | (1\|ID) | 0 | 9 | 556.79 | 71.75 | <0.0001 |
| **2** | **(1\|Mother/ID)** | **1** | **10** | **554.40** | **4.09** | **0.01** |
| 3 | (1 + Test.temp\|ID) | 1 | 10 | 556.98 | 1.81 | 0.08 |
| 4 | (1 + (Test.temp + Test.temp^2^)\|ID) | 2 | 11 | 558.98 | 0.001 | 0.99 |

**Supplemental Table 3.** Different random effects structure for models predicting mean velocity and maximum sustained swimming speed in the **Amazon mollies**. We retained the models that included individual and mother ID for comparison to the Atlantic molly models. Models were specified in the same as above (Table S2).

| M | Random structure | Ref M | M d.f. | AIC | LLR | p-value |
| --- | --- | --- | --- | --- | --- | --- |
| *Mean velocity in an open field (BL/s)* | | | | | | |
| 0 | -- | -- | 8 | 771.97 |  |  |
| 1 | **(1\|ID)** | **0** | **9** | **721.67** | **52.68** | **<0.0001** |
| 2 | (1\|Mother/ID) | 1 | 10 | 723.49 | 0.18 | 0.24 |
| 3 | (1 + Test.temp\|ID) | 1 | 10 | 722.00 | 1.67 | 0.09 |
| 4 | (1 + (Test.temp + Test.temp^2^)\|ID) | 3 | 11 | 724.00 | 0.001 | 0.99 |
| *Maximum sustained swimming speed in a flume (BL/s)* | | | | | | |
| 0 | -- | -- | 8 | 464.57 |  |  |
| 1 | **(1\|ID)** | **0** | **9** | **457.78** | **8.78** | **0.001** |
| 2 | (1\|Mother/ID) | 1 | 10 | 459.55 | 0.22 | 0.23 |
| 3 | (1 + Test.temp\|ID) | 1 | 10 | 459.78 | 0.001 | 0.48 |
| 4 | (1 + (Test.temp + Test.temp^2^)\|ID) | 2 | 11 | 461.78 | 0.001 | 0.99 |

**Supplemental Table 4.** Full results of model testing three-way interactions between species, developmental temperature and acute test temperatures on **locomotor capacity**. We only performed log-likelihood ratio tests to get the overall significance of the three-way interaction terms. Swimming speed and length were centered and scaled to unit variance. Test temperature was centered (but not scaled).

| Effect | Estimate | SE | T-value | LLR | p-value |
| --- | --- | --- | --- | --- | --- |
| Intercept | 0.602 | 0.153 | 3.928 |  |  |
| Length | -0.237 | 0.043 | -5.527 |  |  |
| Observation | 0.031 | 0.016 | 1.841 |  |  |
| Dev.temp:Hot | -0.176 | 0.135 | -1.307 |  |  |
| Test.temp | 0.093 | 0.005 | 16.074 |  |  |
| Species:Atlantic | 0.182 | 0.213 | 0.854 |  |  |
| Test.temp2 | -0.009 | 0.001 | -8.509 |  |  |
| Dev.temp x Test.temp | 0.009 | 0.008 | 1.191 |  |  |
| Dev.temp x Species | -0.482 | 0.205 | -2.348 |  |  |
| Test.temp x Species | -0.045 | 0.008 | -5.412 |  |  |
| Dev.temp x Test.temp2 | 0.0005 | 0.001 | 0.307 |  |  |
| Species x Test.temp2 | -0.001 | 0.002 | -0.987 |  |  |
| Dev.temp x Species x Test.temp | 0.002 | 0.012 | 0.204 | 0.041 | 0.839 |
| **Dev.temp x Species x Test.temp2** | **0.004** | **0.002** | **1.900** | **3.659** | **0.055** |
| Individual | 0.116 |  |  |  |  |
| Mother | 0.054 |  |  |  |  |
| Residual | 0.251 |  |  |  |  |

**Supplemental Table 5**. Full results of model testing three-way interactions between species, developmental temperature and acute test temperatures on **activity in an open field**. Mean velocity and length were centered and scaled to unit variance. Test temperature was centered (but not scaled).

| Effect | Estimate | SE | T-value | LLR | p-value |
| --- | --- | --- | --- | --- | --- |
| Intercept | -0.072 | 0.172 | -0.419 |  |  |
| Length | -0.309 | 0.066 | -4.664 |  |  |
| Observation | -0.047 | 0.021 | -2.212 |  |  |
| Dev.temp:Hot | 0.766 | 0.200 | 3.826 |  |  |
| Test.temp | -0.054 | 0.007 | -6.816 |  |  |
| Species:Atlantic | -0.060 | 0.246 | -0.245 |  |  |
| Test.temp2 | -0.004 | 0.001 | -3.092 |  |  |
| Dev.temp x Test.temp | 0.033 | 0.011 | 3.064 |  |  |
| Dev.temp x Species | -0.448 | 0.302 | -1.481 |  |  |
| Test.temp x Species | 0.064 | 0.011 | 5.689 |  |  |
| Dev.temp x Test.temp2 | -0.002 | 0.002 | -1.300 |  |  |
| Species x Test.temp2 | 0.005 | 0.002 | 2.232 |  |  |
| **Dev.temp x Species x Test.temp** | **-0.032** | **0.015** | **-2.044** | **4.24** | **0.039** |
| Dev.temp x Species x Test.temp2 | 0.0004 | 0.003 | 0.141 | 0.020 | 0.886 |
| Individual | 0.311 |  |  |  |  |
| Mother | 0.035 |  |  |  |  |
| Residual | 0.453 |  |  |  |  |
