# Supplementary material for: Two Locomotor Traits Show Different Patterns of Developmental Plasticity Between Closely Related Clonal and Sexual Fish
Source: Front Physiol. 2021 Oct 12;12:740604. doi: 10.3389/fphys.2021.740604 (PMC8546259; doi:10.3389/fphys.2021.740604)
Supplement: Supplementary file 3 [file Data_Sheet_2.ZIP › DevoPlasticityMollies_210707.html]

DevoPlasticityMollies\_Final


# DevoPlasticityMollies\_Final

#### Kate Laskowski

#### July 7, 2021

“Clonal fish exhibit developmental plasticity in behavioral but not physiological traits” Laskowski, Seebacher, Habedank, Meka & Bierbach

Here we test whether patterns of developmental plasticity in response to early life thermal environments differ between two related traits (locomotor capacity & open field swimming) and whether these patterns differ between two related fish species: the sexual Atlantic molly and the clonal Amazon molly

# DATA INPUT —-

```
library(rmarkdown)
library(plyr)
library(dplyr)
library(ggplot2)
library(MCMCglmm)
library(gridExtra)
library(lme4)
library(nlme)
library(RLRsim)

setwd("C:/Users/katel/Dropbox/IGB work/Bierbach collaborations/Effects of temperature") # kate laptop

# read in data, remove males from mexicana and scale/center variables within each species
data <- read.csv("Laskowski et al_Frontiers_activity and flume_DATA.csv")
data.fem <- filter(data, Sex == 0)
data.fem <- group_by(data.fem, Species) %>% 
  mutate(Mean.velo.BL.scale = as.numeric(scale(Mean.velo.BL))) %>%
  mutate(Ucrit.BL.scale = as.numeric(scale(Ucrit.BL))) %>%
  mutate(Length.scale = as.numeric(scale(Length))) %>%
  mutate(Order.act = Test.day.act-3) %>%
  mutate(Order.swim = Test.day.swim-3) %>%
  mutate(Temp.cen = Test.temp-28)
```

# PRELIMN ANALYSIS - RANDOM STRUCTURE —-

First, we want to determine which random structure best fits our data so we will investigate the inclusion of random intercepts and slopes for individuals and/or mothers for each species and trait

using exactRLRT() to get restricted likelihood ratio test for the different random effects

## > mexicana - activity

```
# ATLANTIC MOLLIES - VOLUNTARY ACTIVITY


data.mex <- filter(data.fem, Species == "mexicana")

# null model
mod0 <- gls(Mean.velo.BL.scale ~ Dev.temp*Temp.cen + Dev.temp*I(Temp.cen^2) + Length.scale +
              Order.act,
             data = data.mex, na.action = na.omit, method = "REML")

# individual only
mod1 <- lmer(Mean.velo.BL.scale ~ Dev.temp*Temp.cen + Dev.temp*I(Temp.cen^2) + Length.scale +
              Order.act + (1|Fish.ID),
             data = data.mex, na.action = na.omit, REML = T)

# mom & individual
mod2 <- lmer(Mean.velo.BL.scale ~ Dev.temp*Temp.cen + Dev.temp*I(Temp.cen^2) + Length.scale +
              Order.act + (1|Mother/Fish.ID),
             data = data.mex, na.action = na.omit, REML = T)

mod2.test <- lmer(Mean.velo.BL.scale ~ Dev.temp*Temp.cen + Dev.temp*I(Temp.cen^2) + Length.scale + Order.act +
                    (1|Mother),
             data = data.mex, na.action = na.omit, REML = T)

# individual slopes
mod3 <- lmer(Mean.velo.BL.scale ~ Dev.temp*Temp.cen + Dev.temp*I(Temp.cen^2) + Length.scale +
              Order.act + (1|Fish.ID) + (0+ Temp.cen|Fish.ID),
             data = data.mex, na.action = na.omit, REML = T)

mod3.test <- lmer(Mean.velo.BL.scale ~ Dev.temp*Temp.cen + Dev.temp*I(Temp.cen^2) + Length.scale + Order.act +
                    (0+ Temp.cen|Fish.ID),
                  data = data.mex, na.action = na.omit, REML = T)
```

```
## boundary (singular) fit: see ?isSingular
```

```
# individual slopes and curves - (model fails to converge)
mod4 <- lmer(Mean.velo.BL.scale ~ Dev.temp*Temp.cen + Dev.temp*I(Temp.cen^2) + Length.scale +
              Order.act + (1|Fish.ID) + (0+ Temp.cen|Fish.ID) + (0 + I(Temp.cen^2)|Fish.ID),
             data = data.mex, na.action = na.omit, REML = T)
```

```
## Warning in checkConv(attr(opt, "derivs"), opt$par, ctrl = control$checkConv, :
## Model failed to converge with max|grad| = 0.086338 (tol = 0.002, component 1)
```

```
mod4.test <- lmer(Mean.velo.BL.scale ~ Dev.temp*Temp.cen + Dev.temp*I(Temp.cen^2) + Length.scale + Order.act +
                    (0 + I(Temp.cen^2)|Fish.ID),
             data = data.mex, na.action = na.omit, REML = T)

AIC(mod0)
```

```
## [1] 738.25
```

```
AIC(mod1)
```

```
## [1] 665.7191
```

```
AIC(mod2)
```

```
## [1] 665.5378
```

```
AIC(mod3)
```

```
## [1] 666.0038
```

```
AIC(mod4)
```

```
## [1] 667.7198
```

```
# test for individual - highly significant
exactRLRT(mod1)
```

```
## 
##  simulated finite sample distribution of RLRT.
##  
##  (p-value based on 10000 simulated values)
## 
## data:  
## RLRT = 74.531, p-value < 2.2e-16
```

```
# test for effect of mom - marginally significant
exactRLRT(mod2.test, mod2, mod1)
```

```
## 
##  simulated finite sample distribution of RLRT.
##  
##  (p-value based on 10000 simulated values)
## 
## data:  
## RLRT = 2.1812, p-value = 0.0479
```

```
# test for addition of slopes - not significant
exactRLRT(mod3.test, mod3, mod1)
```

```
## 
##  simulated finite sample distribution of RLRT.
##  
##  (p-value based on 10000 simulated values)
## 
## data:  
## RLRT = 1.7152, p-value = 0.0871
```

```
# test for addition of curves - not significant
exactRLRT(mod4.test, mod4, mod3)
```

```
## 
##  simulated finite sample distribution of RLRT.
##  
##  (p-value based on 10000 simulated values)
## 
## data:  
## RLRT = 0.284, p-value = 0.2833
```

It appears that the best model both by LRT and AIC (and the fact that the slope model failed dto converge) is the model that includes random intercepts for ID and mother (but not slopes)

## > mexicana - locomotor capacity

```
# ATLANTIC MOLLIES - SWIMMING FLUMES

data.mex <- filter(data.fem, Species == "mexicana")

# null model 
mod0 <- gls(Ucrit.BL.scale  ~ Dev.temp*Temp.cen + Dev.temp*I(Temp.cen^2) + Length.scale + Order.act,
             data = data.mex, na.action = na.omit, method = "REML")

# individual only
mod1 <- lmer(Ucrit.BL.scale  ~ Dev.temp*Temp.cen + Dev.temp*I(Temp.cen^2) + Length.scale + Order.act + (1|Fish.ID), 
             data = data.mex, na.action = na.omit, REML = T)

# mom & individual
mod2 <- lmer(Ucrit.BL.scale  ~ Dev.temp*Temp.cen + Dev.temp*I(Temp.cen^2) + Length.scale + Order.act +
               (1|Mother/Fish.ID),
             data = data.mex, na.action = na.omit, REML = T)

mod2.test <- lmer(Ucrit.BL.scale  ~ Dev.temp*Temp.cen + Dev.temp*I(Temp.cen^2) + Length.scale + Order.act +
                    (1|Mother),
             data = data.mex, na.action = na.omit, REML = T)

# individual slopes
mod3 <- lmer(Ucrit.BL.scale  ~ Dev.temp*Temp.cen + Dev.temp*I(Temp.cen^2) + Length.scale +
              Order.act + (1|Fish.ID) + (0+ Temp.cen|Fish.ID),
             data = data.mex, na.action = na.omit, REML = T)

mod3.test <- lmer(Ucrit.BL.scale  ~ Dev.temp*Temp.cen + Dev.temp*I(Temp.cen^2) + Length.scale + Order.act + (0+ Temp.cen|Fish.ID),
                  data = data.mex, na.action = na.omit, REML = T)
```

```
## boundary (singular) fit: see ?isSingular
```

```
# individual slopes and curves
mod4 <- lmer(Ucrit.BL.scale  ~ Dev.temp*Temp.cen + Dev.temp*I(Temp.cen^2) + Length.scale +
              Order.act + (1|Fish.ID) + (0+ Temp.cen|Fish.ID) + (0 + I(Temp.cen^2)|Fish.ID),
             data = data.mex, na.action = na.omit, REML = T)
```

```
## boundary (singular) fit: see ?isSingular
```

```
mod4.test <- lmer(Ucrit.BL.scale  ~ Dev.temp*Temp.cen + Dev.temp*I(Temp.cen^2) + Length.scale + Order.act +  (0 + I(Temp.cen^2)|Fish.ID),
             data = data.mex, na.action = na.omit, REML = T)


AIC(mod0)
```

```
## [1] 626.5268
```

```
AIC(mod1)
```

```
## [1] 556.4983
```

```
AIC(mod2)
```

```
## [1] 554.4049
```

```
AIC(mod3)
```

```
## [1] 556.8645
```

```
AIC(mod4)
```

```
## [1] 558.8645
```

```
# test for individual - highly significant
exactRLRT(mod1)
```

```
## 
##  simulated finite sample distribution of RLRT.
##  
##  (p-value based on 10000 simulated values)
## 
## data:  
## RLRT = 72.028, p-value < 2.2e-16
```

```
# test for effect of mom - significant
exactRLRT(mod2.test, mod2, mod1)
```

```
## 
##  simulated finite sample distribution of RLRT.
##  
##  (p-value based on 10000 simulated values)
## 
## data:  
## RLRT = 4.0935, p-value = 0.0155
```

```
# test for addition of slopes - marginal (but higher AIC than mod2)
exactRLRT(mod3.test, mod3, mod1)
```

```
## 
##  simulated finite sample distribution of RLRT.
##  
##  (p-value based on 10000 simulated values)
## 
## data:  
## RLRT = 1.6338, p-value = 0.0908
```

```
# test for addition of curves - non-significant
exactRLRT(mod4.test, mod4, mod3)
```

```
## Observed RLRT statistic is 0, no simulation performed.
```

```
## 
##  simulated finite sample distribution of RLRT.
##  
##  (p-value based on 0 simulated values)
## 
## data:  
## RLRT = 0, p-value = 1
```

Again, based on LRT and AIC, the best model appears to be the one with random intercepts for ID and mother

## > formosa - activity

```
# AMAZON MOLLIES - VOLUNTARY ACTIVITY

data.for <- filter(data.fem, Species == "formosa")

# null model 
mod0 <- gls(Mean.velo.BL.scale ~ Dev.temp*Temp.cen + Dev.temp*I(Temp.cen^2) + Length.scale +
              Order.act,
             data = data.for, na.action = na.omit, method = "REML")

# individual only
mod1 <- lmer(Mean.velo.BL.scale ~ Dev.temp*Temp.cen + Dev.temp*I(Temp.cen^2) + Length.scale +
              Order.act + (1|Fish.ID),
             data = data.for, na.action = na.omit, REML = T)

# mom & individual
mod2 <- lmer(Mean.velo.BL.scale ~ Dev.temp*Temp.cen + Dev.temp*I(Temp.cen^2) + Length.scale +
              Order.act + (1|Mother/Fish.ID),
             data = data.for, na.action = na.omit, REML = T)

mod2.test <- lmer(Mean.velo.BL.scale ~ Dev.temp*Temp.cen + Dev.temp*I(Temp.cen^2) + Length.scale +  Order.act + (1|Mother),
             data = data.for, na.action = na.omit, REML = T)

# individual slopes
mod3 <- lmer(Mean.velo.BL.scale ~ Dev.temp*Temp.cen + Dev.temp*I(Temp.cen^2) + Length.scale +
              Order.act + (1|Fish.ID) + (0+ Temp.cen|Fish.ID),
             data = data.for, na.action = na.omit, REML = T)

mod3.test <- lmer(Mean.velo.BL.scale ~ Dev.temp*Temp.cen + Dev.temp*I(Temp.cen^2) + Length.scale +              Order.act + (0+ Temp.cen|Fish.ID),
                  data = data.for, na.action = na.omit, REML = T)
```

```
## boundary (singular) fit: see ?isSingular
```

```
# individual slopes and curves
mod4 <- lmer(Mean.velo.BL.scale ~ Dev.temp*Temp.cen + Dev.temp*I(Temp.cen^2) + Length.scale +
              Order.act + (1|Fish.ID) + (0+ Temp.cen|Fish.ID) + (0 + I(Temp.cen^2)|Fish.ID),
             data = data.for, na.action = na.omit, REML = T)
```

```
## boundary (singular) fit: see ?isSingular
```

```
mod4.test <- lmer(Mean.velo.BL.scale ~ Dev.temp*Temp.cen + Dev.temp*I(Temp.cen^2) + Length.scale + Order.act +  (0 + I(Temp.cen^2)|Fish.ID),
             data = data.for, na.action = na.omit, REML = T)

AIC(mod0)
```

```
## [1] 771.9713
```

```
AIC(mod1)
```

```
## [1] 721.6779
```

```
AIC(mod2)
```

```
## [1] 723.4921
```

```
AIC(mod3)
```

```
## [1] 722.0089
```

```
AIC(mod4)
```

```
## [1] 724.0089
```

```
# test for individual - highly significant
exactRLRT(mod1)
```

```
## 
##  simulated finite sample distribution of RLRT.
##  
##  (p-value based on 10000 simulated values)
## 
## data:  
## RLRT = 52.293, p-value < 2.2e-16
```

```
# test for effect of mom - not really significant
exactRLRT(mod2.test, mod2, mod1)
```

```
## 
##  simulated finite sample distribution of RLRT.
##  
##  (p-value based on 10000 simulated values)
## 
## data:  
## RLRT = 0.1858, p-value = 0.2426
```

```
# test for addition of slopes - marginal (but no decrease in AIC)
exactRLRT(mod3.test, mod3, mod1)
```

```
## 
##  simulated finite sample distribution of RLRT.
##  
##  (p-value based on 10000 simulated values)
## 
## data:  
## RLRT = 1.669, p-value = 0.0912
```

```
# test for addition of curves - not significant
exactRLRT(mod4.test, mod4, mod3)
```

```
## Observed RLRT statistic is 0, no simulation performed.
```

```
## 
##  simulated finite sample distribution of RLRT.
##  
##  (p-value based on 0 simulated values)
## 
## data:  
## RLRT = 0, p-value = 1
```

Based on LRT and AIC, individual intercepts are the ‘best’, but based on the fact that our data in naturally nested in mothers, we will also include intercept for mother

## > formosa - locomotor capacity

```
# AMAZON MOLLIES - SWIMMING FLUMES

data.for <- filter(data.fem, Species == "formosa")

# null model 
mod0 <- gls(Ucrit.BL.scale  ~ Dev.temp*Temp.cen + Dev.temp*I(Temp.cen^2) + Length.scale +
              Order.act,
             data = data.for, na.action = na.omit, method = "REML")

# individual only
mod1 <- lmer(Ucrit.BL.scale  ~ Dev.temp*Temp.cen + Dev.temp*I(Temp.cen^2) + Length.scale +
              Order.act + (1|Fish.ID),
             data = data.for, na.action = na.omit, REML = T)

# mom & individual
mod2 <- lmer(Ucrit.BL.scale  ~ Dev.temp*Temp.cen + Dev.temp*I(Temp.cen^2) + Length.scale +
              Order.act + (1|Mother/Fish.ID),
             data = data.for, na.action = na.omit, REML = T)

mod2.test <- lmer(Ucrit.BL.scale  ~ Dev.temp*Temp.cen + Dev.temp*I(Temp.cen^2) + Length.scale +              Order.act + (1|Mother),
             data = data.for, na.action = na.omit, REML = T)

# individual slopes
mod3 <- lmer(Ucrit.BL.scale  ~ Dev.temp*Temp.cen + Dev.temp*I(Temp.cen^2) + Length.scale +
              Order.act + (1|Fish.ID) + (0+ Temp.cen|Fish.ID),
             data = data.for, na.action = na.omit, REML = T)
```

```
## boundary (singular) fit: see ?isSingular
```

```
mod3.test <- lmer(Ucrit.BL.scale  ~ Dev.temp*Temp.cen + Dev.temp*I(Temp.cen^2) + Length.scale +              Order.act + (0+ Temp.cen|Fish.ID),
                  data = data.for, na.action = na.omit, REML = T)
```

```
## boundary (singular) fit: see ?isSingular
```

```
# individual slopes and curves
mod4 <- lmer(Ucrit.BL.scale  ~ Dev.temp*Temp.cen + Dev.temp*I(Temp.cen^2) + Length.scale +
              Order.act + (1|Fish.ID) + (0+ Temp.cen|Fish.ID) + (0 + I(Temp.cen^2)|Fish.ID),
             data = data.for, na.action = na.omit, REML = T)
```

```
## boundary (singular) fit: see ?isSingular
```

```
mod4.test <- lmer(Ucrit.BL.scale  ~ Dev.temp*Temp.cen + Dev.temp*I(Temp.cen^2) + Length.scale + Order.act +  (0 + I(Temp.cen^2)|Fish.ID),
             data = data.for, na.action = na.omit, REML = T)


AIC(mod0)
```

```
## [1] 464.5724
```

```
AIC(mod1)
```

```
## [1] 457.7842
```

```
AIC(mod2)
```

```
## [1] 459.5582
```

```
AIC(mod3)
```

```
## [1] 459.7842
```

```
AIC(mod4)
```

```
## [1] 461.7842
```

```
# test for individual - highly significant
exactRLRT(mod1)
```

```
## 
##  simulated finite sample distribution of RLRT.
##  
##  (p-value based on 10000 simulated values)
## 
## data:  
## RLRT = 8.7883, p-value = 0.0014
```

```
# test for effect of mom - not significant
exactRLRT(mod2.test, mod2, mod1)
```

```
## 
##  simulated finite sample distribution of RLRT.
##  
##  (p-value based on 10000 simulated values)
## 
## data:  
## RLRT = 0.22591, p-value = 0.228
```

```
# test for addition of slopes - not significant
exactRLRT(mod3.test, mod3, mod1)
```

```
## Observed RLRT statistic is 0, no simulation performed.
```

```
## 
##  simulated finite sample distribution of RLRT.
##  
##  (p-value based on 0 simulated values)
## 
## data:  
## RLRT = 0, p-value = 1
```

```
# test for addition of curves - not significant
exactRLRT(mod4.test, mod4, mod3)
```

```
## 
##  simulated finite sample distribution of RLRT.
##  
##  (p-value based on 10000 simulated values)
## 
## data:  
## RLRT = 1.8048e-10, p-value = 0.4909
```

Based on LRT and AIC, individual intercepts are best, but because data is naturally nested in mother, we will also include mother intercepts

# THREE WAY INTERACTIONS

## > locomotor capacity

```
# First in locomotor capacity (Ucrit.BL)
mod1 <- lmer(Ucrit.BL.scale ~ Length.scale + Order.swim +  Dev.temp*Temp.cen*Species + Dev.temp*I(Temp.cen^2)*Species   
             + (1|Mother/Fish.ID), 
             data = data.fem, na.action = na.omit, REML = T)
plot(mod1)
```

```
qqnorm(resid(mod1))
```

```
summary(mod1)
```

```
## Linear mixed model fit by REML ['lmerMod']
## Formula: Ucrit.BL.scale ~ Length.scale + Order.swim + Dev.temp * Temp.cen *  
##     Species + Dev.temp * I(Temp.cen^2) * Species + (1 | Mother/Fish.ID)
##    Data: data.fem
## 
## REML criterion at convergence: 1011.5
## 
## Scaled residuals: 
##     Min      1Q  Median      3Q     Max 
## -5.1325 -0.5481  0.0183  0.5399  4.0737 
## 
## Random effects:
##  Groups         Name        Variance Std.Dev.
##  Fish.ID:Mother (Intercept) 0.11676  0.3417  
##  Mother         (Intercept) 0.05403  0.2324  
##  Residual                   0.25150  0.5015  
## Number of obs: 536, groups:  Fish.ID:Mother, 109; Mother, 9
## 
## Fixed effects:
##                                             Estimate Std. Error t value
## (Intercept)                                0.6022780  0.1533220   3.928
## Length.scale                              -0.2372559  0.0429269  -5.527
## Order.swim                                 0.0310513  0.0168687   1.841
## Dev.tempHot                               -0.1766374  0.1351886  -1.307
## Temp.cen                                   0.0933768  0.0058090  16.074
## Speciesmexicana                            0.1822140  0.2133638   0.854
## I(Temp.cen^2)                             -0.0095596  0.0011234  -8.509
## Dev.tempHot:Temp.cen                       0.0095292  0.0079990   1.191
## Dev.tempHot:Speciesmexicana               -0.4822381  0.2053490  -2.348
## Temp.cen:Speciesmexicana                  -0.0453223  0.0083749  -5.412
## Dev.tempHot:I(Temp.cen^2)                  0.0004606  0.0015000   0.307
## Speciesmexicana:I(Temp.cen^2)             -0.0015562  0.0015770  -0.987
## Dev.tempHot:Temp.cen:Speciesmexicana       0.0024118  0.0118248   0.204
## Dev.tempHot:Speciesmexicana:I(Temp.cen^2)  0.0041951  0.0022085   1.900
```

```
## 
## Correlation matrix not shown by default, as p = 14 > 12.
## Use print(x, correlation=TRUE)  or
##     vcov(x)        if you need it
```

### … Species x Dev.Temp x TT2

```
# LRT for overall significance of Dev.temp*Species*TT2
# first re-run model with ML
mod1 <- lmer(Ucrit.BL.scale ~ Length.scale + Order.swim +  Dev.temp*Temp.cen*Species +
               Dev.temp*I(Temp.cen^2)*Species   
             + (1|Mother/Fish.ID), 
             data = data.fem, na.action = na.omit, REML = F)

mod2 <- lmer(Ucrit.BL.scale ~ Length.scale + Order.swim +  Dev.temp*Temp.cen*Species +   
               Dev.temp*I(Temp.cen^2)+ Dev.temp*Species + Species*I(Temp.cen^2)
             + (1|Mother/Fish.ID), 
             data = data.fem, na.action = na.omit, REML = F)
anova(mod1, mod2)
```

```
## Data: data.fem
## Models:
## mod2: Ucrit.BL.scale ~ Length.scale + Order.swim + Dev.temp * Temp.cen * 
## mod2:     Species + Dev.temp * I(Temp.cen^2) + Dev.temp * Species + 
## mod2:     Species * I(Temp.cen^2) + (1 | Mother/Fish.ID)
## mod1: Ucrit.BL.scale ~ Length.scale + Order.swim + Dev.temp * Temp.cen * 
## mod1:     Species + Dev.temp * I(Temp.cen^2) * Species + (1 | Mother/Fish.ID)
##      Df    AIC    BIC  logLik deviance  Chisq Chi Df Pr(>Chisq)  
## mod2 16 945.41 1014.0 -456.70   913.41                           
## mod1 17 943.75 1016.6 -454.87   909.75 3.6591      1    0.05576 .
## ---
## Signif. codes:  0 '***' 0.001 '**' 0.01 '*' 0.05 '.' 0.1 ' ' 1
```

### … Species x Dev.Temp x TT

```
# Dev.temp*Species*TT
mod3 <- lmer(Ucrit.BL.scale ~ Length.scale + Order.swim +  
               Dev.temp*Temp.cen + Dev.temp*Species + Temp.cen*Species + 
               Dev.temp*I(Temp.cen^2)*Species   
             + (1|Mother/Fish.ID), 
             data = data.fem, na.action = na.omit, REML = F)
anova(mod1, mod3)
```

```
## Data: data.fem
## Models:
## mod3: Ucrit.BL.scale ~ Length.scale + Order.swim + Dev.temp * Temp.cen + 
## mod3:     Dev.temp * Species + Temp.cen * Species + Dev.temp * I(Temp.cen^2) * 
## mod3:     Species + (1 | Mother/Fish.ID)
## mod1: Ucrit.BL.scale ~ Length.scale + Order.swim + Dev.temp * Temp.cen * 
## mod1:     Species + Dev.temp * I(Temp.cen^2) * Species + (1 | Mother/Fish.ID)
##      Df    AIC    BIC  logLik deviance Chisq Chi Df Pr(>Chisq)
## mod3 16 941.79 1010.3 -454.90   909.79                        
## mod1 17 943.75 1016.6 -454.87   909.75 0.041      1     0.8395
```

## > Unforced activity

```
# Then in activity in an open field

mod4 <- lmer(Mean.velo.BL.scale ~ Length.scale + Order.act +  Dev.temp*Temp.cen*Species +
               Dev.temp*I(Temp.cen^2)*Species   
             + (1|Mother/Fish.ID), 
             data = data.fem, na.action = na.omit, REML = T)
summary(mod4)
```

```
## Linear mixed model fit by REML ['lmerMod']
## Formula: Mean.velo.BL.scale ~ Length.scale + Order.act + Dev.temp * Temp.cen *  
##     Species + Dev.temp * I(Temp.cen^2) * Species + (1 | Mother/Fish.ID)
##    Data: data.fem
## 
## REML criterion at convergence: 1340.9
## 
## Scaled residuals: 
##     Min      1Q  Median      3Q     Max 
## -2.7207 -0.5928 -0.0890  0.5323  4.1082 
## 
## Random effects:
##  Groups         Name        Variance Std.Dev.
##  Fish.ID:Mother (Intercept) 0.31151  0.5581  
##  Mother         (Intercept) 0.03505  0.1872  
##  Residual                   0.45345  0.6734  
## Number of obs: 535, groups:  Fish.ID:Mother, 108; Mother, 9
## 
## Fixed effects:
##                                             Estimate Std. Error t value
## (Intercept)                               -0.0721954  0.1722964  -0.419
## Length.scale                              -0.3091381  0.0662752  -4.664
## Order.act                                 -0.0474153  0.0214392  -2.212
## Dev.tempHot                                0.7660853  0.2002304   3.826
## Temp.cen                                  -0.0541308  0.0079413  -6.816
## Speciesmexicana                           -0.0602376  0.2463255  -0.245
## I(Temp.cen^2)                             -0.0044897  0.0014519  -3.092
## Dev.tempHot:Temp.cen                       0.0332032  0.0108359   3.064
## Dev.tempHot:Speciesmexicana               -0.4482996  0.3026529  -1.481
## Temp.cen:Speciesmexicana                   0.0649459  0.0114158   5.689
## Dev.tempHot:I(Temp.cen^2)                 -0.0026184  0.0020137  -1.300
## Speciesmexicana:I(Temp.cen^2)              0.0046529  0.0020846   2.232
## Dev.tempHot:Temp.cen:Speciesmexicana      -0.0323806  0.0158447  -2.044
## Dev.tempHot:Speciesmexicana:I(Temp.cen^2)  0.0004162  0.0029535   0.141
```

```
## 
## Correlation matrix not shown by default, as p = 14 > 12.
## Use print(x, correlation=TRUE)  or
##     vcov(x)        if you need it
```

### … Species x Dev.Temp x TT2

```
# overall significance of Dev.temp*Species*TT2 interaction
# first re-run model in ML
mod4 <- lmer(Mean.velo.BL.scale ~ Length.scale + Order.act +  Dev.temp*Temp.cen*Species +
               Dev.temp*I(Temp.cen^2)*Species   
             + (1|Mother/Fish.ID), 
             data = data.fem, na.action = na.omit, REML = F)

mod5 <- lmer(Mean.velo.BL.scale ~ Length.scale + Order.act +  Dev.temp*Temp.cen*Species +
               Dev.temp*I(Temp.cen^2) + Dev.temp*Species + I(Temp.cen^2)*Species +  
             + (1|Mother/Fish.ID), 
             data = data.fem, na.action = na.omit, REML = F)
anova(mod4, mod5)
```

```
## Data: data.fem
## Models:
## mod5: Mean.velo.BL.scale ~ Length.scale + Order.act + Dev.temp * Temp.cen * 
## mod5:     Species + Dev.temp * I(Temp.cen^2) + Dev.temp * Species + 
## mod5:     I(Temp.cen^2) * Species + +(1 | Mother/Fish.ID)
## mod4: Mean.velo.BL.scale ~ Length.scale + Order.act + Dev.temp * Temp.cen * 
## mod4:     Species + Dev.temp * I(Temp.cen^2) * Species + (1 | Mother/Fish.ID)
##      Df  AIC    BIC  logLik deviance  Chisq Chi Df Pr(>Chisq)
## mod5 16 1279 1347.5 -623.49     1247                         
## mod4 17 1281 1353.8 -623.48     1247 0.0204      1     0.8864
```

### … Species x Dev. Temp x TT

```
# Dev.temp*Species*TT interaction
mod6 <- lmer(Mean.velo.BL.scale ~ Length.scale + Order.act +  
               Dev.temp*Temp.cen + Dev.temp*Species + Temp.cen*Species +
               Dev.temp*I(Temp.cen^2)*Species   
             + (1|Mother/Fish.ID), 
             data = data.fem, na.action = na.omit, REML = F)
anova(mod4, mod6)
```

```
## Data: data.fem
## Models:
## mod6: Mean.velo.BL.scale ~ Length.scale + Order.act + Dev.temp * Temp.cen + 
## mod6:     Dev.temp * Species + Temp.cen * Species + Dev.temp * I(Temp.cen^2) * 
## mod6:     Species + (1 | Mother/Fish.ID)
## mod4: Mean.velo.BL.scale ~ Length.scale + Order.act + Dev.temp * Temp.cen * 
## mod4:     Species + Dev.temp * I(Temp.cen^2) * Species + (1 | Mother/Fish.ID)
##      Df    AIC    BIC  logLik deviance Chisq Chi Df Pr(>Chisq)  
## mod6 16 1283.2 1351.7 -625.60   1251.2                          
## mod4 17 1281.0 1353.8 -623.48   1247.0 4.242      1    0.03943 *
## ---
## Signif. codes:  0 '***' 0.001 '**' 0.01 '*' 0.05 '.' 0.1 ' ' 1
```

# SPECIES - SPECIFIC EFFECTS

## > mexicana - locomoter capacity

```
data <- read.csv("Both species_combined data_analysis.csv")
data.fem <- filter(data, Sex == 0)
data.fem <- group_by(data.fem, Species) %>% 
  mutate(Mean.velo.BL.scale = as.numeric(scale(Mean.velo.BL))) %>%
  mutate(Ucrit.BL.scale = as.numeric(scale(Ucrit.BL))) %>%
  mutate(Length.scale = as.numeric(scale(Length))) %>%
  mutate(Order.act = Test.day.act-3) %>%
  mutate(Order.swim = Test.day.swim-3) %>%
  mutate(Temp.cen = Test.temp-28)
data.mex <- filter(data.fem, Species == "mexicana")

# full model with ML
mex.ucrit1 <- lmer(Ucrit.BL.scale ~ Length.scale + Order.swim +  Dev.temp*Temp.cen + Dev.temp*I(Temp.cen^2)   
             + (1|Mother/Fish.ID), 
             data = data.mex, na.action = na.omit, REML = F)
summary(mex.ucrit1)
```

```
## Linear mixed model fit by maximum likelihood  ['lmerMod']
## Formula: Ucrit.BL.scale ~ Length.scale + Order.swim + Dev.temp * Temp.cen +  
##     Dev.temp * I(Temp.cen^2) + (1 | Mother/Fish.ID)
##    Data: data.mex
## 
##      AIC      BIC   logLik deviance df.resid 
##    503.0    541.6   -240.5    481.0      236 
## 
## Scaled residuals: 
##     Min      1Q  Median      3Q     Max 
## -5.0492 -0.5353 -0.0581  0.5077  3.6332 
## 
## Random effects:
##  Groups         Name        Variance Std.Dev.
##  Fish.ID:Mother (Intercept) 0.21048  0.4588  
##  Mother         (Intercept) 0.06968  0.2640  
##  Residual                   0.29509  0.5432  
## Number of obs: 247, groups:  Fish.ID:Mother, 50; Mother, 5
## 
## Fixed effects:
##                            Estimate Std. Error t value
## (Intercept)                0.790208   0.177885   4.442
## Length.scale              -0.232898   0.087989  -2.647
## Order.swim                -0.007989   0.025280  -0.316
## Dev.tempHot               -0.657428   0.197237  -3.333
## Temp.cen                   0.050022   0.006647   7.525
## I(Temp.cen^2)             -0.011272   0.001218  -9.255
## Dev.tempHot:Temp.cen       0.010860   0.009443   1.150
## Dev.tempHot:I(Temp.cen^2)  0.004727   0.001755   2.693
## 
## Correlation of Fixed Effects:
##             (Intr) Lngth. Ordr.s Dv.tmH Tmp.cn I(T.^2 D.H:T.
## Length.scal  0.255                                          
## Order.swim  -0.028  0.004                                   
## Dev.tempHot -0.530 -0.409  0.010                            
## Temp.cen     0.003  0.000 -0.198  0.000                     
## I(Tmp.cn^2) -0.370  0.001  0.077  0.333 -0.001              
## Dv.tmpHt:T.  0.000 -0.004  0.062 -0.004 -0.689 -0.005       
## D.H:I(T.^2)  0.256 -0.003 -0.037 -0.480 -0.003 -0.692  0.015
```

test Dev.temp:TT2 interaction

```
# LLR tests to estimate overall significance of fixed effects

mex.ucrit2 <-  lmer(Ucrit.BL.scale ~ Length.scale + Order.swim  + Dev.temp*Temp.cen + Dev.temp + I(Temp.cen^2)  
              + (1|Mother/Fish.ID),  
              data = data.mex, na.action = na.omit, REML = F)

anova(mex.ucrit1, mex.ucrit2)
```

```
## Data: data.mex
## Models:
## mex.ucrit2: Ucrit.BL.scale ~ Length.scale + Order.swim + Dev.temp * Temp.cen + 
## mex.ucrit2:     Dev.temp + I(Temp.cen^2) + (1 | Mother/Fish.ID)
## mex.ucrit1: Ucrit.BL.scale ~ Length.scale + Order.swim + Dev.temp * Temp.cen + 
## mex.ucrit1:     Dev.temp * I(Temp.cen^2) + (1 | Mother/Fish.ID)
##            Df    AIC    BIC  logLik deviance  Chisq Chi Df Pr(>Chisq)   
## mex.ucrit2 10 508.09 543.19 -244.05   488.09                            
## mex.ucrit1 11 502.97 541.57 -240.48   480.97 7.1271      1   0.007593 **
## ---
## Signif. codes:  0 '***' 0.001 '**' 0.01 '*' 0.05 '.' 0.1 ' ' 1
```

test Dev.temp:TT interaction

```
mex.ucrit3 <- lmer(Ucrit.BL.scale ~ Length.scale + Order.swim  +  Temp.cen + Dev.temp*I(Temp.cen^2)   
             + (1|Mother/Fish.ID),  
             data = data.mex, na.action = na.omit, REML = F)

anova(mex.ucrit1, mex.ucrit3)
```

```
## Data: data.mex
## Models:
## mex.ucrit3: Ucrit.BL.scale ~ Length.scale + Order.swim + Temp.cen + Dev.temp * 
## mex.ucrit3:     I(Temp.cen^2) + (1 | Mother/Fish.ID)
## mex.ucrit1: Ucrit.BL.scale ~ Length.scale + Order.swim + Dev.temp * Temp.cen + 
## mex.ucrit1:     Dev.temp * I(Temp.cen^2) + (1 | Mother/Fish.ID)
##            Df    AIC    BIC  logLik deviance  Chisq Chi Df Pr(>Chisq)
## mex.ucrit3 10 502.29 537.38 -241.14   482.29                         
## mex.ucrit1 11 502.97 541.57 -240.48   480.97 1.3187      1     0.2508
```

test main effect of Test.cen

```
mex.ucrit4 <-  lmer(Ucrit.BL.scale ~ Length.scale + Order.swim  + Dev.temp*I(Temp.cen^2) 
              + (1 |Mother/Fish.ID),  
              data = data.mex, na.action = na.omit, REML = F)
anova(mex.ucrit3, mex.ucrit4)
```

```
## Data: data.mex
## Models:
## mex.ucrit4: Ucrit.BL.scale ~ Length.scale + Order.swim + Dev.temp * I(Temp.cen^2) + 
## mex.ucrit4:     (1 | Mother/Fish.ID)
## mex.ucrit3: Ucrit.BL.scale ~ Length.scale + Order.swim + Temp.cen + Dev.temp * 
## mex.ucrit3:     I(Temp.cen^2) + (1 | Mother/Fish.ID)
##            Df    AIC    BIC  logLik deviance  Chisq Chi Df Pr(>Chisq)    
## mex.ucrit4  9 601.36 632.94 -291.68   583.36                             
## mex.ucrit3 10 502.29 537.38 -241.14   482.29 101.07      1  < 2.2e-16 ***
## ---
## Signif. codes:  0 '***' 0.001 '**' 0.01 '*' 0.05 '.' 0.1 ' ' 1
```

test main effect of length

```
mex.ucrit5 <-  lmer(Ucrit.BL.scale ~Order.swim  +  Temp.cen + Dev.temp*I(Temp.cen^2)  
              + (1 |Mother/Fish.ID),  
              data = data.mex, na.action = na.omit, REML = F)

anova(mex.ucrit3, mex.ucrit5)
```

```
## Data: data.mex
## Models:
## mex.ucrit5: Ucrit.BL.scale ~ Order.swim + Temp.cen + Dev.temp * I(Temp.cen^2) + 
## mex.ucrit5:     (1 | Mother/Fish.ID)
## mex.ucrit3: Ucrit.BL.scale ~ Length.scale + Order.swim + Temp.cen + Dev.temp * 
## mex.ucrit3:     I(Temp.cen^2) + (1 | Mother/Fish.ID)
##            Df    AIC    BIC  logLik deviance  Chisq Chi Df Pr(>Chisq)  
## mex.ucrit5  9 506.29 537.88 -244.15   488.29                           
## mex.ucrit3 10 502.29 537.38 -241.14   482.29 6.0079      1    0.01424 *
## ---
## Signif. codes:  0 '***' 0.001 '**' 0.01 '*' 0.05 '.' 0.1 ' ' 1
```

test main effect of observation

```
mex.ucrit6 <-  lmer(Ucrit.BL.scale ~ Length.scale   +  Temp.cen + Dev.temp*I(Temp.cen^2)   
              + (1|Mother/Fish.ID),  
              data = data.mex, na.action = na.omit, REML = F)
anova(mex.ucrit3, mex.ucrit6)
```

```
## Data: data.mex
## Models:
## mex.ucrit6: Ucrit.BL.scale ~ Length.scale + Temp.cen + Dev.temp * I(Temp.cen^2) + 
## mex.ucrit6:     (1 | Mother/Fish.ID)
## mex.ucrit3: Ucrit.BL.scale ~ Length.scale + Order.swim + Temp.cen + Dev.temp * 
## mex.ucrit3:     I(Temp.cen^2) + (1 | Mother/Fish.ID)
##            Df    AIC    BIC  logLik deviance  Chisq Chi Df Pr(>Chisq)
## mex.ucrit6  9 500.43 532.02 -241.22   482.43                         
## mex.ucrit3 10 502.29 537.38 -241.14   482.29 0.1494      1     0.6991
```

Full model with REML to report estimates

```
mex.ucrit.final <- lmer(Ucrit.BL.scale ~ Length.scale + Order.swim +  Dev.temp*Temp.cen + Dev.temp*I(Temp.cen^2)   
             + (1|Mother/Fish.ID), 
             data = data.mex, na.action = na.omit, REML = T)
summary(mex.ucrit.final)
```

```
## Linear mixed model fit by REML ['lmerMod']
## Formula: Ucrit.BL.scale ~ Length.scale + Order.swim + Dev.temp * Temp.cen +  
##     Dev.temp * I(Temp.cen^2) + (1 | Mother/Fish.ID)
##    Data: data.mex
## 
## REML criterion at convergence: 532.7
## 
## Scaled residuals: 
##     Min      1Q  Median      3Q     Max 
## -5.0028 -0.5247 -0.0579  0.4993  3.5681 
## 
## Random effects:
##  Groups         Name        Variance Std.Dev.
##  Fish.ID:Mother (Intercept) 0.2195   0.4686  
##  Mother         (Intercept) 0.1035   0.3217  
##  Residual                   0.3028   0.5503  
## Number of obs: 247, groups:  Fish.ID:Mother, 50; Mother, 5
## 
## Fixed effects:
##                            Estimate Std. Error t value
## (Intercept)                0.802662   0.198363   4.046
## Length.scale              -0.222303   0.090662  -2.452
## Order.swim                -0.007997   0.025608  -0.312
## Dev.tempHot               -0.680323   0.202072  -3.367
## Temp.cen                   0.050008   0.006733   7.427
## I(Temp.cen^2)             -0.011274   0.001234  -9.139
## Dev.tempHot:Temp.cen       0.010879   0.009565   1.137
## Dev.tempHot:I(Temp.cen^2)  0.004730   0.001778   2.660
## 
## Correlation of Fixed Effects:
##             (Intr) Lngth. Ordr.s Dv.tmH Tmp.cn I(T.^2 D.H:T.
## Length.scal  0.243                                          
## Order.swim  -0.025  0.004                                   
## Dev.tempHot -0.487 -0.416  0.010                            
## Temp.cen     0.003  0.000 -0.198  0.000                     
## I(Tmp.cn^2) -0.336  0.001  0.077  0.329 -0.001              
## Dv.tmpHt:T.  0.000 -0.003  0.062 -0.004 -0.689 -0.005       
## D.H:I(T.^2)  0.233 -0.003 -0.037 -0.475 -0.003 -0.692  0.015
```

```
0.212/(0.212+.100+0.293)
```

```
## [1] 0.3504132
```

to estimate R2 values from mixed model after Nakagawa & Schielzeth 2013 MEE

marginal R2

```
Fixed <- fixef(mex.ucrit.final)[2]*model.matrix(mex.ucrit.final)[,2] + fixef(mex.ucrit.final)[3]*model.matrix(mex.ucrit.final)[,3] + 
  fixef(mex.ucrit.final)[4]*model.matrix(mex.ucrit.final)[,4] + fixef(mex.ucrit.final)[5]*model.matrix(mex.ucrit.final)[,5] + 
  fixef(mex.ucrit.final)[6]*model.matrix(mex.ucrit.final)[,6] + fixef(mex.ucrit.final)[7]*model.matrix(mex.ucrit.final)[,7] + 
  fixef(mex.ucrit.final)[8]*model.matrix(mex.ucrit.final)[,8] 

varF <- var(Fixed)

varF/(varF + VarCorr(mex.ucrit.final)$Fish.ID[1] + VarCorr(mex.ucrit.final)$Mother[1]+
        attr(VarCorr(mex.ucrit.final), "sc")^2)
```

```
## [1] 0.4114536
```

conditional R2

```
(varF + VarCorr(mex.ucrit.final)$Fish.ID[1] + VarCorr(mex.ucrit.final)$Mother[1])/
  (varF + VarCorr(mex.ucrit.final)$Fish.ID[1] +  VarCorr(mex.ucrit.final)$Mother[1]+
     (attr(VarCorr(mex.ucrit.final), "sc")^2))
```

```
## [1] 0.7152691
```

## > mexicana - activity

```
data <- read.csv("Both species_combined data_analysis.csv")
data.fem <- filter(data, Sex == 0)
data.fem <- group_by(data.fem, Species) %>% 
  mutate(Mean.velo.BL.scale = as.numeric(scale(Mean.velo.BL))) %>%
  mutate(Ucrit.BL.scale = as.numeric(scale(Ucrit.BL))) %>%
  mutate(Length.scale = as.numeric(scale(Length))) %>%
  mutate(Order.act = Test.day.act-3) %>%
  mutate(Order.swim = Test.day.swim-3) %>%
  mutate(Temp.cen = Test.temp-28)
data.mex <- filter(data.fem, Species == "mexicana")


# full model with ML
mex.act1 <- lmer(Mean.velo.BL.scale ~ Length.scale + Order.act +  Dev.temp*Temp.cen + Dev.temp*I(Temp.cen^2)   
             + (1|Mother/Fish.ID)  , 
             data = data.mex, na.action = na.omit, REML = F)
```

Dev.temp:TT2 interaction

```
# LLR tests for main effects


mex.act2 <-  lmer(Mean.velo.BL.scale ~ Length.scale + Order.act  + Dev.temp*Temp.cen + Dev.temp + I(Temp.cen^2)  
              + (1|Mother/Fish.ID)  ,  
              data = data.mex, na.action = na.omit, REML = F)

anova(mex.act1, mex.act2)
```

```
## Data: data.mex
## Models:
## mex.act2: Mean.velo.BL.scale ~ Length.scale + Order.act + Dev.temp * Temp.cen + 
## mex.act2:     Dev.temp + I(Temp.cen^2) + (1 | Mother/Fish.ID)
## mex.act1: Mean.velo.BL.scale ~ Length.scale + Order.act + Dev.temp * Temp.cen + 
## mex.act1:     Dev.temp * I(Temp.cen^2) + (1 | Mother/Fish.ID)
##          Df    AIC    BIC logLik deviance  Chisq Chi Df Pr(>Chisq)
## mex.act2 10 615.61 650.82 -297.8   595.61                         
## mex.act1 11 616.60 655.33 -297.3   594.60 1.0128      1     0.3142
```

test Dev.temp:TT interaction

```
mex.act3 <- lmer(Mean.velo.BL.scale ~ Length.scale + Order.act  + Dev.temp + Temp.cen + Dev.temp*I(Temp.cen^2)  
             + (1|Mother/Fish.ID)  ,  
             data = data.mex, na.action = na.omit, REML = F)
```

```
## Warning in checkConv(attr(opt, "derivs"), opt$par, ctrl = control$checkConv, :
## Model failed to converge with max|grad| = 0.00369595 (tol = 0.002, component 1)
```

```
anova(mex.act1, mex.act3)
```

```
## Data: data.mex
## Models:
## mex.act3: Mean.velo.BL.scale ~ Length.scale + Order.act + Dev.temp + Temp.cen + 
## mex.act3:     Dev.temp * I(Temp.cen^2) + (1 | Mother/Fish.ID)
## mex.act1: Mean.velo.BL.scale ~ Length.scale + Order.act + Dev.temp * Temp.cen + 
## mex.act1:     Dev.temp * I(Temp.cen^2) + (1 | Mother/Fish.ID)
##          Df   AIC    BIC logLik deviance  Chisq Chi Df Pr(>Chisq)
## mex.act3 10 614.6 649.82 -297.3    594.6                         
## mex.act1 11 616.6 655.33 -297.3    594.6 0.0048      1     0.9446
```

test main effect of TT2 (can remove both 2-way interactions)

```
mex.act4 <- lmer(Mean.velo.BL.scale ~ Length.scale + Order.act  + Dev.temp + Temp.cen  + I(Temp.cen^2)  
             + (1|Mother/Fish.ID)  ,  
             data = data.mex, na.action = na.omit, REML = F)

mex.act5 <-  lmer(Mean.velo.BL.scale ~ Length.scale + Order.act  + Dev.temp + Temp.cen    
              + (1|Mother/Fish.ID)  ,  
              data = data.mex, na.action = na.omit, REML = F)
anova(mex.act4, mex.act5)
```

```
## Data: data.mex
## Models:
## mex.act5: Mean.velo.BL.scale ~ Length.scale + Order.act + Dev.temp + Temp.cen + 
## mex.act5:     (1 | Mother/Fish.ID)
## mex.act4: Mean.velo.BL.scale ~ Length.scale + Order.act + Dev.temp + Temp.cen + 
## mex.act4:     I(Temp.cen^2) + (1 | Mother/Fish.ID)
##          Df    AIC    BIC  logLik deviance Chisq Chi Df Pr(>Chisq)
## mex.act5  8 612.27 640.45 -298.14   596.27                        
## mex.act4  9 613.61 645.31 -297.81   595.61  0.66      1     0.4165
```

test main effect of length

```
mex.act6 <-  lmer(Mean.velo.BL.scale ~ Order.act  + Dev.temp + Temp.cen + I(Temp.cen^2)  
              + (1|Mother/Fish.ID) ,  
              data = data.mex, na.action = na.omit, REML = F)

anova(mex.act4, mex.act6)
```

```
## Data: data.mex
## Models:
## mex.act6: Mean.velo.BL.scale ~ Order.act + Dev.temp + Temp.cen + I(Temp.cen^2) + 
## mex.act6:     (1 | Mother/Fish.ID)
## mex.act4: Mean.velo.BL.scale ~ Length.scale + Order.act + Dev.temp + Temp.cen + 
## mex.act4:     I(Temp.cen^2) + (1 | Mother/Fish.ID)
##          Df    AIC    BIC  logLik deviance  Chisq Chi Df Pr(>Chisq)   
## mex.act6  8 621.21 649.38 -302.61   605.21                            
## mex.act4  9 613.61 645.31 -297.81   595.61 9.5993      1   0.001947 **
## ---
## Signif. codes:  0 '***' 0.001 '**' 0.01 '*' 0.05 '.' 0.1 ' ' 1
```

test main effect of observation

```
mex.act7 <-  lmer(Mean.velo.BL.scale ~ Length.scale   + Dev.temp + Temp.cen +  I(Temp.cen^2)  
              + (1|Mother/Fish.ID)  ,  
              data = data.mex, na.action = na.omit, REML = F)
anova(mex.act4, mex.act7)
```

```
## Data: data.mex
## Models:
## mex.act7: Mean.velo.BL.scale ~ Length.scale + Dev.temp + Temp.cen + I(Temp.cen^2) + 
## mex.act7:     (1 | Mother/Fish.ID)
## mex.act4: Mean.velo.BL.scale ~ Length.scale + Order.act + Dev.temp + Temp.cen + 
## mex.act4:     I(Temp.cen^2) + (1 | Mother/Fish.ID)
##          Df    AIC    BIC  logLik deviance  Chisq Chi Df Pr(>Chisq)  
## mex.act7  8 614.79 642.96 -299.39   598.79                           
## mex.act4  9 613.61 645.31 -297.81   595.61 3.1726      1    0.07488 .
## ---
## Signif. codes:  0 '***' 0.001 '**' 0.01 '*' 0.05 '.' 0.1 ' ' 1
```

test main effect of Dev.temp

```
mex.act8 <- lmer(Mean.velo.BL.scale ~ Length.scale + Order.act  + Temp.cen +  I(Temp.cen^2)  
             + (1|Mother/Fish.ID)  ,  
             data = data.mex, na.action = na.omit, REML = F)
anova(mex.act4, mex.act8)
```

```
## Data: data.mex
## Models:
## mex.act8: Mean.velo.BL.scale ~ Length.scale + Order.act + Temp.cen + I(Temp.cen^2) + 
## mex.act8:     (1 | Mother/Fish.ID)
## mex.act4: Mean.velo.BL.scale ~ Length.scale + Order.act + Dev.temp + Temp.cen + 
## mex.act4:     I(Temp.cen^2) + (1 | Mother/Fish.ID)
##          Df    AIC    BIC  logLik deviance  Chisq Chi Df Pr(>Chisq)
## mex.act8  8 612.83 641.00 -298.42   596.83                         
## mex.act4  9 613.61 645.31 -297.81   595.61 1.2181      1     0.2697
```

test main effect of test.temp

```
mex.act9 <- lmer(Mean.velo.BL.scale ~ Length.scale + Order.act  + Dev.temp  +  I(Temp.cen^2)  
             + (1|Mother/Fish.ID) ,  
             data = data.mex, na.action = na.omit, REML = F)
anova(mex.act4, mex.act9)
```

```
## Data: data.mex
## Models:
## mex.act9: Mean.velo.BL.scale ~ Length.scale + Order.act + Dev.temp + I(Temp.cen^2) + 
## mex.act9:     (1 | Mother/Fish.ID)
## mex.act4: Mean.velo.BL.scale ~ Length.scale + Order.act + Dev.temp + Temp.cen + 
## mex.act4:     I(Temp.cen^2) + (1 | Mother/Fish.ID)
##          Df    AIC    BIC  logLik deviance  Chisq Chi Df Pr(>Chisq)  
## mex.act9  8 615.38 643.55 -299.69   599.38                           
## mex.act4  9 613.61 645.31 -297.81   595.61 3.7656      1    0.05232 .
## ---
## Signif. codes:  0 '***' 0.001 '**' 0.01 '*' 0.05 '.' 0.1 ' ' 1
```

# full model with REML to report estimates

```
mex.act.final <- lmer(Mean.velo.BL.scale ~ Length.scale + Order.act +  Dev.temp*Temp.cen + Dev.temp*I(Temp.cen^2)   
             + (1|Mother/Fish.ID), 
             data = data.mex, na.action = na.omit, REML = T)
summary(mex.act.final)
```

```
## Linear mixed model fit by REML ['lmerMod']
## Formula: Mean.velo.BL.scale ~ Length.scale + Order.act + Dev.temp * Temp.cen +  
##     Dev.temp * I(Temp.cen^2) + (1 | Mother/Fish.ID)
##    Data: data.mex
## 
## REML criterion at convergence: 643.5
## 
## Scaled residuals: 
##      Min       1Q   Median       3Q      Max 
## -2.31918 -0.62750 -0.08252  0.54705  2.63730 
## 
## Random effects:
##  Groups         Name        Variance Std.Dev.
##  Fish.ID:Mother (Intercept) 0.38139  0.6176  
##  Mother         (Intercept) 0.05503  0.2346  
##  Residual                   0.46424  0.6814  
## Number of obs: 250, groups:  Fish.ID:Mother, 50; Mother, 5
## 
## Fixed effects:
##                             Estimate Std. Error t value
## (Intercept)               -0.1552163  0.2002928  -0.775
## Length.scale              -0.3573847  0.1135932  -3.146
## Order.act                 -0.0531212  0.0311508  -1.705
## Dev.tempHot                0.3520069  0.2540701   1.385
## Temp.cen                   0.0110346  0.0081903   1.347
## I(Temp.cen^2)              0.0001620  0.0015135   0.107
## Dev.tempHot:Temp.cen       0.0008024  0.0116950   0.069
## Dev.tempHot:I(Temp.cen^2) -0.0021777  0.0021886  -0.995
## 
## Correlation of Fixed Effects:
##             (Intr) Lngth. Ordr.c Dv.tmH Tmp.cn I(T.^2 D.H:T.
## Length.scal  0.272                                          
## Order.act   -0.002  0.000                                   
## Dev.tempHot -0.604 -0.397  0.029                            
## Temp.cen     0.000  0.000 -0.146 -0.004                     
## I(Tmp.cn^2) -0.411  0.000  0.004  0.324 -0.001              
## Dv.tmpHt:T.  0.000  0.000  0.009  0.000 -0.687  0.000       
## D.H:I(T.^2)  0.284  0.000 -0.061 -0.469  0.009 -0.692 -0.001
```

```
0.367/(0.381+0.053+0.446)
```

```
## [1] 0.4170455
```

Estimate R2

Marginal R2

```
Fixed <- fixef(mex.act.final)[2]*model.matrix(mex.act.final)[,2] + fixef(mex.act.final)[3]*model.matrix(mex.act.final)[,3] + 
  fixef(mex.act.final)[4]*model.matrix(mex.act.final)[,4] + fixef(mex.act.final)[5]*model.matrix(mex.act.final)[,5] + 
  fixef(mex.act.final)[6]*model.matrix(mex.act.final)[,6] + fixef(mex.act.final)[7]*model.matrix(mex.act.final)[,7] + 
  fixef(mex.act.final)[8]*model.matrix(mex.act.final)[,8] 

varF <- var(Fixed)

# marginal R2
varF/(varF + VarCorr(mex.act.final)$Fish.ID[1] +   VarCorr(mex.act.final)$Mother[1] +
        (attr(VarCorr(mex.act.final), "sc")^2))
```

```
## [1] 0.1186206
```

Conditional R2

```
(varF + VarCorr(mex.act.final)$Fish.ID[1] + VarCorr(mex.act.final)$Mother[1])/
  (varF + VarCorr(mex.act.final)$Fish.ID[1] +   VarCorr(mex.act.final)$Mother[1] +
     (attr(VarCorr(mex.act.final), "sc")^2))
```

```
## [1] 0.5456964
```

## > mexicana - correlation

```
# MCMCglmm is hte best package to look at multivariate covariances
# if both traits at scaled to unit variance prior to analysis then
# resulting covariances are equivalent to correlations
data <- read.csv("Both species_combined data_analysis.csv")
data.fem <- filter(data, Sex == 0)
data.fem <- group_by(data.fem, Species) %>% 
  mutate(Mean.velo.BL.scale = as.numeric(scale(Mean.velo.BL))) %>%
  mutate(Ucrit.BL.scale = as.numeric(scale(Ucrit.BL))) %>%
  mutate(Length.scale = as.numeric(scale(Length))) %>%
  mutate(Order.act = Test.day.act-3) %>%
  mutate(Order.swim = Test.day.swim-3) %>%
  mutate(Temp.cen = Test.temp-28)
data.mex <- filter(data.fem, Species == "mexicana")

# MCMCglmm needs data as "data.frame"
data.mex2 <- data.frame(data.mex)

# parameter expanded priors
prior.multi <- list(R = list(V = diag(2), nu = 2.02), 
                    G = list(G1 =list(V=diag(2), nu = 2.02, alpha.mu = rep(0,2),  alpha.V = diag(2)*24*2)))


# covariance between velocity and Ucrit for both temperatures
velo.ucrit.mex <- MCMCglmm(cbind(Mean.velo.BL.scale, Ucrit.BL.scale) ~ 1,
                            random = ~us(trait):Fish.ID,
                            prior = prior.multi, 
                            data = data.mex2,
                            rcov = ~ us(trait):units, 
                            family = c("gaussian", "gaussian"), 
                            nitt = 400000, thin = 200, burnin = 1000, verbose = F)
summary(velo.ucrit.mex)
```

```
## 
##  Iterations = 1001:399801
##  Thinning interval  = 200
##  Sample size  = 1995 
## 
##  DIC: 1205.108 
## 
##  G-structure:  ~us(trait):Fish.ID
## 
##                                                         post.mean l-95% CI
## traitMean.velo.BL.scale:traitMean.velo.BL.scale.Fish.ID    0.5684  0.32419
## traitUcrit.BL.scale:traitMean.velo.BL.scale.Fish.ID        0.2573  0.08638
## traitMean.velo.BL.scale:traitUcrit.BL.scale.Fish.ID        0.2573  0.08638
## traitUcrit.BL.scale:traitUcrit.BL.scale.Fish.ID            0.3525  0.17593
##                                                         u-95% CI eff.samp
## traitMean.velo.BL.scale:traitMean.velo.BL.scale.Fish.ID   0.8444     1995
## traitUcrit.BL.scale:traitMean.velo.BL.scale.Fish.ID       0.4448     1995
## traitMean.velo.BL.scale:traitUcrit.BL.scale.Fish.ID       0.4448     1995
## traitUcrit.BL.scale:traitUcrit.BL.scale.Fish.ID           0.5631     1995
## 
##  R-structure:  ~us(trait):units
## 
##                                                       post.mean l-95% CI
## traitMean.velo.BL.scale:traitMean.velo.BL.scale.units   0.48180  0.39407
## traitUcrit.BL.scale:traitMean.velo.BL.scale.units       0.02188 -0.05909
## traitMean.velo.BL.scale:traitUcrit.BL.scale.units       0.02188 -0.05909
## traitUcrit.BL.scale:traitUcrit.BL.scale.units           0.68635  0.56230
##                                                       u-95% CI eff.samp
## traitMean.velo.BL.scale:traitMean.velo.BL.scale.units   0.5750     1995
## traitUcrit.BL.scale:traitMean.velo.BL.scale.units       0.1038     2219
## traitMean.velo.BL.scale:traitUcrit.BL.scale.units       0.1038     2219
## traitUcrit.BL.scale:traitUcrit.BL.scale.units           0.8239     2158
## 
##  Location effects: cbind(Mean.velo.BL.scale, Ucrit.BL.scale) ~ 1 
## 
##              post.mean   l-95% CI   u-95% CI eff.samp pMCMC
## (Intercept)  0.0007645 -0.1775023  0.1821376     1995 0.996
```

Only for Warm fish

```
hot.velo.ucrit.mex <- MCMCglmm(cbind(Mean.velo.BL.scale, Ucrit.BL.scale) ~ 1,
                           random = ~us(trait):Fish.ID,
                           prior = prior.multi, 
                           data = data.mex2[which(data.mex2$Dev.temp == "Hot"),],
                           rcov = ~ us(trait):units, 
                           family = c("gaussian", "gaussian"), 
                           nitt = 400000, thin = 200, burnin = 1000, verbose = F)
summary(hot.velo.ucrit.mex)
```

```
## 
##  Iterations = 1001:399801
##  Thinning interval  = 200
##  Sample size  = 1995 
## 
##  DIC: 503.5472 
## 
##  G-structure:  ~us(trait):Fish.ID
## 
##                                                         post.mean   l-95% CI
## traitMean.velo.BL.scale:traitMean.velo.BL.scale.Fish.ID  0.443712  1.716e-01
## traitUcrit.BL.scale:traitMean.velo.BL.scale.Fish.ID     -0.005383 -1.157e-01
## traitMean.velo.BL.scale:traitUcrit.BL.scale.Fish.ID     -0.005383 -1.157e-01
## traitUcrit.BL.scale:traitUcrit.BL.scale.Fish.ID          0.063022  4.045e-08
##                                                         u-95% CI eff.samp
## traitMean.velo.BL.scale:traitMean.velo.BL.scale.Fish.ID   0.8026     1995
## traitUcrit.BL.scale:traitMean.velo.BL.scale.Fish.ID       0.1032     1816
## traitMean.velo.BL.scale:traitUcrit.BL.scale.Fish.ID       0.1032     1816
## traitUcrit.BL.scale:traitUcrit.BL.scale.Fish.ID           0.1803     1995
## 
##  R-structure:  ~us(trait):units
## 
##                                                       post.mean l-95% CI
## traitMean.velo.BL.scale:traitMean.velo.BL.scale.units   0.40405   0.3046
## traitUcrit.BL.scale:traitMean.velo.BL.scale.units       0.06066  -0.0365
## traitMean.velo.BL.scale:traitUcrit.BL.scale.units       0.06066  -0.0365
## traitUcrit.BL.scale:traitUcrit.BL.scale.units           0.49097   0.3539
##                                                       u-95% CI eff.samp
## traitMean.velo.BL.scale:traitMean.velo.BL.scale.units   0.5187     1995
## traitUcrit.BL.scale:traitMean.velo.BL.scale.units       0.1517     1995
## traitMean.velo.BL.scale:traitUcrit.BL.scale.units       0.1517     1995
## traitUcrit.BL.scale:traitUcrit.BL.scale.units           0.6294     1995
## 
##  Location effects: cbind(Mean.velo.BL.scale, Ucrit.BL.scale) ~ 1 
## 
##             post.mean l-95% CI u-95% CI eff.samp pMCMC  
## (Intercept)   -0.1864  -0.3424  -0.0517     1995 0.019 *
## ---
## Signif. codes:  0 '***' 0.001 '**' 0.01 '*' 0.05 '.' 0.1 ' ' 1
```

only for cold fish

```
cold.velo.ucrit.mex <- MCMCglmm(cbind(Mean.velo.BL.scale, Ucrit.BL.scale) ~ 1,
                           random = ~us(trait):Fish.ID,
                           prior = prior.multi, 
                           data = data.mex2[which(data.mex2$Dev.temp == "Cold"),],
                           rcov = ~ us(trait):units, 
                           family = c("gaussian", "gaussian"), 
                           nitt = 400000, thin = 200, burnin = 1000, verbose = F)
summary(cold.velo.ucrit.mex)
```

```
## 
##  Iterations = 1001:399801
##  Thinning interval  = 200
##  Sample size  = 1995 
## 
##  DIC: 684.9226 
## 
##  G-structure:  ~us(trait):Fish.ID
## 
##                                                         post.mean l-95% CI
## traitMean.velo.BL.scale:traitMean.velo.BL.scale.Fish.ID    0.8226   0.3850
## traitUcrit.BL.scale:traitMean.velo.BL.scale.Fish.ID        0.4899   0.1709
## traitMean.velo.BL.scale:traitUcrit.BL.scale.Fish.ID        0.4899   0.1709
## traitUcrit.BL.scale:traitUcrit.BL.scale.Fish.ID            0.5533   0.1748
##                                                         u-95% CI eff.samp
## traitMean.velo.BL.scale:traitMean.velo.BL.scale.Fish.ID   1.4236     1995
## traitUcrit.BL.scale:traitMean.velo.BL.scale.Fish.ID       0.9425     2254
## traitMean.velo.BL.scale:traitUcrit.BL.scale.Fish.ID       0.9425     2254
## traitUcrit.BL.scale:traitUcrit.BL.scale.Fish.ID           1.0078     1995
## 
##  R-structure:  ~us(trait):units
## 
##                                                       post.mean l-95% CI
## traitMean.velo.BL.scale:traitMean.velo.BL.scale.units  0.576018   0.4362
## traitUcrit.BL.scale:traitMean.velo.BL.scale.units     -0.009385  -0.1639
## traitMean.velo.BL.scale:traitUcrit.BL.scale.units     -0.009385  -0.1639
## traitUcrit.BL.scale:traitUcrit.BL.scale.units          0.900825   0.6780
##                                                       u-95% CI eff.samp
## traitMean.velo.BL.scale:traitMean.velo.BL.scale.units   0.7436     1995
## traitUcrit.BL.scale:traitMean.velo.BL.scale.units       0.1197     1935
## traitMean.velo.BL.scale:traitUcrit.BL.scale.units       0.1197     1935
## traitUcrit.BL.scale:traitUcrit.BL.scale.units           1.1500     1995
## 
##  Location effects: cbind(Mean.velo.BL.scale, Ucrit.BL.scale) ~ 1 
## 
##             post.mean l-95% CI u-95% CI eff.samp pMCMC
## (Intercept)    0.1389  -0.1796   0.4653     1995 0.386
```

no real difference between the two treatments

## > formosa - locomotor capacity

```
data <- read.csv("Both species_combined data_analysis.csv")
data.fem <- filter(data, Sex == 0)
data.fem <- group_by(data.fem, Species) %>% 
  mutate(Mean.velo.BL.scale = as.numeric(scale(Mean.velo.BL))) %>%
  mutate(Ucrit.BL.scale = as.numeric(scale(Ucrit.BL))) %>%
  mutate(Length.scale = as.numeric(scale(Length))) %>%
  mutate(Order.act = Test.day.act-3) %>%
  mutate(Order.swim = Test.day.swim-3) %>%
  mutate(Temp.cen = Test.temp-28)
data.for <- filter(data.fem, Species == "formosa")


# full model with ML
for.ucrit1 <- lmer(Ucrit.BL.scale ~ Length.scale + Order.swim +  Dev.temp*Temp.cen + Dev.temp*I(Temp.cen^2)   
             + (1|Mother/Fish.ID), 
             data = data.for, na.action = na.omit, REML = F)
```

Testing Dev.temp \* TT^2 interaction

```
for.ucrit2 <- lmer(Ucrit.BL.scale ~ Length.scale + Order.swim +  Dev.temp*Temp.cen + Dev.temp + I(Temp.cen^2)   
             + (1|Mother/Fish.ID), 
             data = data.for, na.action = na.omit, REML = F)
```

```
## Warning in checkConv(attr(opt, "derivs"), opt$par, ctrl = control$checkConv, :
## Model failed to converge with max|grad| = 0.00455427 (tol = 0.002, component 1)
```

```
anova(for.ucrit1, for.ucrit2)
```

```
## Data: data.for
## Models:
## for.ucrit2: Ucrit.BL.scale ~ Length.scale + Order.swim + Dev.temp * Temp.cen + 
## for.ucrit2:     Dev.temp + I(Temp.cen^2) + (1 | Mother/Fish.ID)
## for.ucrit1: Ucrit.BL.scale ~ Length.scale + Order.swim + Dev.temp * Temp.cen + 
## for.ucrit1:     Dev.temp * I(Temp.cen^2) + (1 | Mother/Fish.ID)
##            Df    AIC    BIC  logLik deviance  Chisq Chi Df Pr(>Chisq)
## for.ucrit2 10 407.27 443.93 -193.63   387.27                         
## for.ucrit1 11 409.07 449.40 -193.54   387.07 0.1941      1     0.6596
```

testing Dev.temp \* TT interaction

```
for.ucrit3 <- lmer(Ucrit.BL.scale ~ Length.scale + Order.swim +  Dev.temp + Temp.cen + Dev.temp*I(Temp.cen^2)   
             + (1|Mother/Fish.ID), 
             data = data.for, na.action = na.omit, REML = F)
anova(for.ucrit1, for.ucrit3)
```

```
## Data: data.for
## Models:
## for.ucrit3: Ucrit.BL.scale ~ Length.scale + Order.swim + Dev.temp + Temp.cen + 
## for.ucrit3:     Dev.temp * I(Temp.cen^2) + (1 | Mother/Fish.ID)
## for.ucrit1: Ucrit.BL.scale ~ Length.scale + Order.swim + Dev.temp * Temp.cen + 
## for.ucrit1:     Dev.temp * I(Temp.cen^2) + (1 | Mother/Fish.ID)
##            Df    AIC    BIC  logLik deviance  Chisq Chi Df Pr(>Chisq)
## for.ucrit3 10 408.80 445.47 -194.40   388.80                         
## for.ucrit1 11 409.07 449.40 -193.54   387.07 1.7287      1     0.1886
```

testing main effect of TT2 (can remove both 2-way interactions)

```
for.ucrit4 <- lmer(Ucrit.BL.scale ~ Length.scale + Order.swim +  Dev.temp + Temp.cen + I(Temp.cen^2)   
             + (1|Mother/Fish.ID), 
             data = data.for, na.action = na.omit, REML = F)

for.ucrit5 <- lmer(Ucrit.BL.scale ~ Length.scale + Order.swim +  Dev.temp + Temp.cen    
             + (1|Mother/Fish.ID), 
             data = data.for, na.action = na.omit, REML = F)
anova(for.ucrit4, for.ucrit5)
```

```
## Data: data.for
## Models:
## for.ucrit5: Ucrit.BL.scale ~ Length.scale + Order.swim + Dev.temp + Temp.cen + 
## for.ucrit5:     (1 | Mother/Fish.ID)
## for.ucrit4: Ucrit.BL.scale ~ Length.scale + Order.swim + Dev.temp + Temp.cen + 
## for.ucrit4:     I(Temp.cen^2) + (1 | Mother/Fish.ID)
##            Df    AIC    BIC  logLik deviance  Chisq Chi Df Pr(>Chisq)    
## for.ucrit5  8 501.93 531.26 -242.97   485.93                             
## for.ucrit4  9 406.99 439.99 -194.50   388.99 96.941      1  < 2.2e-16 ***
## ---
## Signif. codes:  0 '***' 0.001 '**' 0.01 '*' 0.05 '.' 0.1 ' ' 1
```

effect of test.temp

```
for.ucrit6 <- lmer(Ucrit.BL.scale ~ Length.scale + Order.swim +  Dev.temp  + I(Temp.cen^2)   
             + (1|Mother/Fish.ID), 
             data = data.for, na.action = na.omit, REML = F)
```

```
## boundary (singular) fit: see ?isSingular
```

```
anova(for.ucrit4, for.ucrit6)
```

```
## Data: data.for
## Models:
## for.ucrit6: Ucrit.BL.scale ~ Length.scale + Order.swim + Dev.temp + I(Temp.cen^2) + 
## for.ucrit6:     (1 | Mother/Fish.ID)
## for.ucrit4: Ucrit.BL.scale ~ Length.scale + Order.swim + Dev.temp + Temp.cen + 
## for.ucrit4:     I(Temp.cen^2) + (1 | Mother/Fish.ID)
##            Df    AIC    BIC  logLik deviance  Chisq Chi Df Pr(>Chisq)    
## for.ucrit6  8 750.91 780.24 -367.45   734.91                             
## for.ucrit4  9 406.99 439.99 -194.50   388.99 345.92      1  < 2.2e-16 ***
## ---
## Signif. codes:  0 '***' 0.001 '**' 0.01 '*' 0.05 '.' 0.1 ' ' 1
```

effect of Dev.temp

```
for.ucrit7 <- lmer(Ucrit.BL.scale ~ Length.scale + Order.swim  + Temp.cen + I(Temp.cen^2)   
             + (1|Mother/Fish.ID), 
             data = data.for, na.action = na.omit, REML = F)
```

```
## Warning in checkConv(attr(opt, "derivs"), opt$par, ctrl = control$checkConv, :
## Model failed to converge with max|grad| = 0.00742198 (tol = 0.002, component 1)
```

```
anova(for.ucrit7, for.ucrit4)
```

```
## Data: data.for
## Models:
## for.ucrit7: Ucrit.BL.scale ~ Length.scale + Order.swim + Temp.cen + I(Temp.cen^2) + 
## for.ucrit7:     (1 | Mother/Fish.ID)
## for.ucrit4: Ucrit.BL.scale ~ Length.scale + Order.swim + Dev.temp + Temp.cen + 
## for.ucrit4:     I(Temp.cen^2) + (1 | Mother/Fish.ID)
##            Df    AIC    BIC  logLik deviance  Chisq Chi Df Pr(>Chisq)  
## for.ucrit7  8 409.35 438.68 -196.67   393.35                           
## for.ucrit4  9 406.99 439.99 -194.50   388.99 4.3562      1    0.03687 *
## ---
## Signif. codes:  0 '***' 0.001 '**' 0.01 '*' 0.05 '.' 0.1 ' ' 1
```

effect of observation

```
for.ucrit8 <- lmer(Ucrit.BL.scale ~ Length.scale  +  Dev.temp + Temp.cen + I(Temp.cen^2)   
             + (1|Mother/Fish.ID), 
             data = data.for, na.action = na.omit, REML = F)
anova(for.ucrit4, for.ucrit8)
```

```
## Data: data.for
## Models:
## for.ucrit8: Ucrit.BL.scale ~ Length.scale + Dev.temp + Temp.cen + I(Temp.cen^2) + 
## for.ucrit8:     (1 | Mother/Fish.ID)
## for.ucrit4: Ucrit.BL.scale ~ Length.scale + Order.swim + Dev.temp + Temp.cen + 
## for.ucrit4:     I(Temp.cen^2) + (1 | Mother/Fish.ID)
##            Df    AIC    BIC  logLik deviance Chisq Chi Df Pr(>Chisq)    
## for.ucrit8  8 416.78 446.11 -200.39   400.78                            
## for.ucrit4  9 406.99 439.99 -194.50   388.99 11.79      1  0.0005956 ***
## ---
## Signif. codes:  0 '***' 0.001 '**' 0.01 '*' 0.05 '.' 0.1 ' ' 1
```

effect of body length

```
for.ucrit9 <- lmer(Ucrit.BL.scale ~  Order.swim +  Dev.temp + Temp.cen + I(Temp.cen^2)   
             + (1|Mother/Fish.ID), 
             data = data.for, na.action = na.omit, REML = F)
anova(for.ucrit4, for.ucrit9)
```

```
## Data: data.for
## Models:
## for.ucrit9: Ucrit.BL.scale ~ Order.swim + Dev.temp + Temp.cen + I(Temp.cen^2) + 
## for.ucrit9:     (1 | Mother/Fish.ID)
## for.ucrit4: Ucrit.BL.scale ~ Length.scale + Order.swim + Dev.temp + Temp.cen + 
## for.ucrit4:     I(Temp.cen^2) + (1 | Mother/Fish.ID)
##            Df    AIC    BIC logLik deviance  Chisq Chi Df Pr(>Chisq)    
## for.ucrit9  8 441.80 471.13 -212.9   425.80                             
## for.ucrit4  9 406.99 439.99 -194.5   388.99 36.807      1  1.304e-09 ***
## ---
## Signif. codes:  0 '***' 0.001 '**' 0.01 '*' 0.05 '.' 0.1 ' ' 1
```

Full model with REML for reporting estimates

```
for.ucrit.final <- lmer(Ucrit.BL.scale ~ Length.scale + Order.swim +  Dev.temp*Temp.cen + Dev.temp*I(Temp.cen^2)   
             + (1|Mother/Fish.ID), 
             data = data.for, na.action = na.omit, REML = T)
summary(for.ucrit.final)
```

```
## Linear mixed model fit by REML ['lmerMod']
## Formula: Ucrit.BL.scale ~ Length.scale + Order.swim + Dev.temp * Temp.cen +  
##     Dev.temp * I(Temp.cen^2) + (1 | Mother/Fish.ID)
##    Data: data.for
## 
## REML criterion at convergence: 447.5
## 
## Scaled residuals: 
##     Min      1Q  Median      3Q     Max 
## -2.7819 -0.5889  0.1110  0.6405  2.1783 
## 
## Random effects:
##  Groups         Name        Variance Std.Dev.
##  Fish.ID:Mother (Intercept) 0.033680 0.1835  
##  Mother         (Intercept) 0.002034 0.0451  
##  Residual                   0.202757 0.4503  
## Number of obs: 289, groups:  Fish.ID:Mother, 59; Mother, 4
## 
## Fixed effects:
##                             Estimate Std. Error t value
## (Intercept)                0.5616545  0.0803293   6.992
## Length.scale              -0.2482694  0.0358433  -6.927
## Order.swim                 0.0756301  0.0218834   3.456
## Dev.tempHot               -0.1834285  0.1027471  -1.785
## Temp.cen                   0.0926280  0.0052235  17.733
## I(Temp.cen^2)             -0.0088324  0.0010428  -8.470
## Dev.tempHot:Temp.cen       0.0093671  0.0071813   1.304
## Dev.tempHot:I(Temp.cen^2)  0.0005881  0.0013472   0.437
## 
## Correlation of Fixed Effects:
##             (Intr) Lngth. Ordr.s Dv.tmH Tmp.cn I(T.^2 D.H:T.
## Length.scal -0.013                                          
## Order.swim  -0.242  0.018                                   
## Dev.tempHot -0.664  0.010 -0.030                            
## Temp.cen     0.012 -0.001 -0.078  0.008                     
## I(Tmp.cn^2) -0.707  0.006  0.351  0.476 -0.014              
## Dv.tmpHt:T.  0.006 -0.004 -0.003 -0.001 -0.723 -0.010       
## D.H:I(T.^2)  0.471 -0.004  0.043 -0.717 -0.013 -0.663  0.001
```

```
0.0197/(0.0197+0.001+0.119)
```

```
## [1] 0.1410165
```

estimating R2

Marginal R2

```
Fixed <- fixef(for.ucrit.final)[2]*model.matrix(for.ucrit.final)[,2] + fixef(for.ucrit.final)[3]*model.matrix(for.ucrit.final)[,3] + 
  fixef(for.ucrit.final)[4]*model.matrix(for.ucrit.final)[,4] + fixef(for.ucrit.final)[5]*model.matrix(for.ucrit.final)[,5] + 
  fixef(for.ucrit.final)[6]*model.matrix(for.ucrit.final)[,6] + fixef(for.ucrit.final)[7]*model.matrix(for.ucrit.final)[,7] + 
  fixef(for.ucrit.final)[8]*model.matrix(for.ucrit.final)[,8] 

varF <- var(Fixed)

# marginal R2
varF/(varF + VarCorr(for.ucrit.final)$Fish.ID[1] + VarCorr(for.ucrit.final)$Mother[1] +
        attr(VarCorr(for.ucrit.final), "sc")^2)
```

```
## [1] 0.7626951
```

conditional R2

```
(varF + VarCorr(for.ucrit.final)$Fish.ID[1]+VarCorr(for.ucrit.final)$Mother[1] )/
  (varF + VarCorr(for.ucrit.final)$Fish.ID[1] + VarCorr(for.ucrit.final)$Mother[1] +
     (attr(VarCorr(for.ucrit.final), "sc")^2))
```

```
## [1] 0.7982343
```

## > formosa - activity

```
data <- read.csv("Both species_combined data_analysis.csv")
data.fem <- filter(data, Sex == 0)
data.fem <- group_by(data.fem, Species) %>% 
  mutate(Mean.velo.BL.scale = as.numeric(scale(Mean.velo.BL))) %>%
  mutate(Ucrit.BL.scale = as.numeric(scale(Ucrit.BL))) %>%
  mutate(Length.scale = as.numeric(scale(Length))) %>%
  mutate(Order.act = Test.day.act-3) %>%
  mutate(Order.swim = Test.day.swim-3) %>%
  mutate(Temp.cen = Test.temp-28)
data.for <- filter(data.fem, Species == "formosa")

# full model with ML
for.act1 <- lmer(Mean.velo.BL.scale ~ Length.scale + Order.act +  Dev.temp*Temp.cen + Dev.temp*I(Temp.cen^2)   
             + (1|Mother/Fish.ID), 
             data = data.for, na.action = na.omit, REML = F)
```

testing for Dev.temp \* TT2 interaction

```
for.act2 <-  lmer(Mean.velo.BL.scale ~ Length.scale + Order.act +  Dev.temp*Temp.cen + Dev.temp + I(Temp.cen^2)   
             + (1|Mother/Fish.ID), 
             data = data.for, na.action = na.omit, REML = F)
anova(for.act1, for.act2)
```

```
## Data: data.for
## Models:
## for.act2: Mean.velo.BL.scale ~ Length.scale + Order.act + Dev.temp * Temp.cen + 
## for.act2:     Dev.temp + I(Temp.cen^2) + (1 | Mother/Fish.ID)
## for.act1: Mean.velo.BL.scale ~ Length.scale + Order.act + Dev.temp * Temp.cen + 
## for.act1:     Dev.temp * I(Temp.cen^2) + (1 | Mother/Fish.ID)
##          Df    AIC    BIC  logLik deviance  Chisq Chi Df Pr(>Chisq)
## for.act2 10 671.32 707.85 -325.66   651.32                         
## for.act1 11 671.56 711.73 -324.78   649.56 1.7674      1     0.1837
```

testing for Dev.temp \* TT interaction

```
for.act3 <-  lmer(Mean.velo.BL.scale ~ Length.scale + Order.act +  Dev.temp + Temp.cen + Dev.temp*I(Temp.cen^2)   
             + (1|Mother/Fish.ID), 
             data = data.for, na.action = na.omit, REML = F)
anova(for.act1, for.act3)
```

```
## Data: data.for
## Models:
## for.act3: Mean.velo.BL.scale ~ Length.scale + Order.act + Dev.temp + Temp.cen + 
## for.act3:     Dev.temp * I(Temp.cen^2) + (1 | Mother/Fish.ID)
## for.act1: Mean.velo.BL.scale ~ Length.scale + Order.act + Dev.temp * Temp.cen + 
## for.act1:     Dev.temp * I(Temp.cen^2) + (1 | Mother/Fish.ID)
##          Df    AIC    BIC  logLik deviance  Chisq Chi Df Pr(>Chisq)   
## for.act3 10 679.01 715.54 -329.51   659.01                            
## for.act1 11 671.56 711.73 -324.78   649.56 9.4586      1   0.002102 **
## ---
## Signif. codes:  0 '***' 0.001 '**' 0.01 '*' 0.05 '.' 0.1 ' ' 1
```

testing for main effect of TT2

```
for.act4 <-  lmer(Mean.velo.BL.scale ~ Length.scale + Order.act +  Dev.temp*Temp.cen     
             + (1|Mother/Fish.ID), 
             data = data.for, na.action = na.omit, REML = F)

anova(for.act2, for.act4)
```

```
## Data: data.for
## Models:
## for.act4: Mean.velo.BL.scale ~ Length.scale + Order.act + Dev.temp * Temp.cen + 
## for.act4:     (1 | Mother/Fish.ID)
## for.act2: Mean.velo.BL.scale ~ Length.scale + Order.act + Dev.temp * Temp.cen + 
## for.act2:     Dev.temp + I(Temp.cen^2) + (1 | Mother/Fish.ID)
##          Df    AIC    BIC  logLik deviance  Chisq Chi Df Pr(>Chisq)    
## for.act4  9 701.72 734.59 -341.86   683.72                             
## for.act2 10 671.32 707.85 -325.66   651.32 32.396      1  1.257e-08 ***
## ---
## Signif. codes:  0 '***' 0.001 '**' 0.01 '*' 0.05 '.' 0.1 ' ' 1
```

main effect of observation

```
for.act5 <- lmer(Mean.velo.BL.scale ~ Length.scale +  Dev.temp*Temp.cen + I(Temp.cen^2)   
             + (1|Fish.ID), 
             data = data.for, na.action = na.omit, REML = F)
anova(for.act2, for.act5)
```

```
## Data: data.for
## Models:
## for.act5: Mean.velo.BL.scale ~ Length.scale + Dev.temp * Temp.cen + I(Temp.cen^2) + 
## for.act5:     (1 | Fish.ID)
## for.act2: Mean.velo.BL.scale ~ Length.scale + Order.act + Dev.temp * Temp.cen + 
## for.act2:     Dev.temp + I(Temp.cen^2) + (1 | Mother/Fish.ID)
##          Df    AIC    BIC  logLik deviance  Chisq Chi Df Pr(>Chisq)
## for.act5  8 669.40 698.62 -326.70   653.40                         
## for.act2 10 671.32 707.85 -325.66   651.32 2.0757      2     0.3542
```

effect of length

```
for.act6 <- lmer(Mean.velo.BL.scale ~ Order.act +  Dev.temp*Temp.cen + I(Temp.cen^2)   
             + (1|Mother/Fish.ID), 
             data = data.for, na.action = na.omit, REML = F)
anova(for.act2, for.act6)
```

```
## Data: data.for
## Models:
## for.act6: Mean.velo.BL.scale ~ Order.act + Dev.temp * Temp.cen + I(Temp.cen^2) + 
## for.act6:     (1 | Mother/Fish.ID)
## for.act2: Mean.velo.BL.scale ~ Length.scale + Order.act + Dev.temp * Temp.cen + 
## for.act2:     Dev.temp + I(Temp.cen^2) + (1 | Mother/Fish.ID)
##          Df    AIC    BIC  logLik deviance  Chisq Chi Df Pr(>Chisq)    
## for.act6  9 680.91 713.78 -331.45   662.91                             
## for.act2 10 671.32 707.85 -325.66   651.32 11.582      1   0.000666 ***
## ---
## Signif. codes:  0 '***' 0.001 '**' 0.01 '*' 0.05 '.' 0.1 ' ' 1
```

full model with REML for reporting estimates

```
for.act.final <- lmer(Mean.velo.BL.scale ~ Length.scale + Order.act +  Dev.temp*Temp.cen + Dev.temp*I(Temp.cen^2)   
             + (1|Mother/Fish.ID), 
             data = data.for, na.action = na.omit, REML = T)
summary(for.act.final)
```

```
## Linear mixed model fit by REML ['lmerMod']
## Formula: Mean.velo.BL.scale ~ Length.scale + Order.act + Dev.temp * Temp.cen +  
##     Dev.temp * I(Temp.cen^2) + (1 | Mother/Fish.ID)
##    Data: data.for
## 
## REML criterion at convergence: 701.5
## 
## Scaled residuals: 
##     Min      1Q  Median      3Q     Max 
## -2.7415 -0.5201 -0.0786  0.5041  4.1919 
## 
## Random effects:
##  Groups         Name        Variance Std.Dev.
##  Fish.ID:Mother (Intercept) 0.257540 0.50748 
##  Mother         (Intercept) 0.009171 0.09576 
##  Residual                   0.445848 0.66772 
## Number of obs: 285, groups:  Fish.ID:Mother, 58; Mother, 4
## 
## Fixed effects:
##                            Estimate Std. Error t value
## (Intercept)               -0.073479   0.144692  -0.508
## Length.scale              -0.275981   0.079416  -3.475
## Order.act                 -0.042092   0.029619  -1.421
## Dev.tempHot                0.762516   0.189713   4.019
## Temp.cen                  -0.053720   0.008003  -6.712
## I(Temp.cen^2)             -0.004496   0.001440  -3.121
## Dev.tempHot:Temp.cen       0.033066   0.010749   3.076
## Dev.tempHot:I(Temp.cen^2) -0.002627   0.001997  -1.316
## 
## Correlation of Fixed Effects:
##             (Intr) Lngth. Ordr.c Dv.tmH Tmp.cn I(T.^2 D.H:T.
## Length.scal -0.015                                          
## Order.act    0.019  0.004                                   
## Dev.tempHot -0.674  0.028  0.002                            
## Temp.cen    -0.002  0.002  0.257  0.006                     
## I(Tmp.cn^2) -0.539  0.000 -0.045  0.410  0.004              
## Dv.tmpHt:T.  0.004  0.001 -0.042 -0.010 -0.706 -0.010       
## D.H:I(T.^2)  0.388  0.001 -0.005 -0.573 -0.013 -0.720  0.013
```

```
0.207/(0.207+0.007+0.358)
```

```
## [1] 0.3618881
```

Estimating R2

marginal R2

```
Fixed <- fixef(for.act.final)[2]*model.matrix(for.act.final)[,2] + fixef(for.act.final)[3]*model.matrix(for.act.final)[,3] + 
  fixef(for.act.final)[4]*model.matrix(for.act.final)[,4] + fixef(for.act.final)[5]*model.matrix(for.act.final)[,5] + 
  fixef(for.act.final)[6]*model.matrix(for.act.final)[,6] + fixef(for.act.final)[7]*model.matrix(for.act.final)[,7] + 
  fixef(for.act.final)[8]*model.matrix(for.act.final)[,8] 

varF <- var(Fixed)

varF/(varF + VarCorr(for.act.final)$Fish.ID[1] + VarCorr(for.act.final)$Mother[1] +
        attr(VarCorr(for.act.final), "sc")^2)
```

```
## [1] 0.3047242
```

conditional R2

```
(varF + VarCorr(for.act.final)$Fish.ID[1] + VarCorr(for.act.final)$Mother[1])/
  (varF + VarCorr(for.act.final)$Fish.ID[1] +  VarCorr(for.act.final)$Mother[1] +
     (attr(VarCorr(for.act.final), "sc")^2))
```

```
## [1] 0.5649662
```

## > formosa - correlation

```
data <- read.csv("Both species_combined data_analysis.csv")
data.fem <- filter(data, Sex == 0)
data.fem <- group_by(data.fem, Species) %>% 
  mutate(Mean.velo.BL.scale = as.numeric(scale(Mean.velo.BL))) %>%
  mutate(Ucrit.BL.scale = as.numeric(scale(Ucrit.BL))) %>%
  mutate(Length.scale = as.numeric(scale(Length))) %>%
  mutate(Order.act = Test.day.act-3) %>%
  mutate(Order.swim = Test.day.swim-3) %>%
  mutate(Temp.cen = Test.temp-28)
data.for <- filter(data.fem, Species == "formosa")

# MCMCglmm needs data as "data.frame"
data.for2 <- data.frame(data.for)

# parameter expanded priors
prior.multi <- list(R = list(V = diag(2), nu = 2.02), 
                    G = list(G1 =list(V=diag(2), nu = 2.02, alpha.mu = rep(0,2),  alpha.V = diag(2)*24*2)))


# covariance between velocity and Ucrit for both temperatures
velo.ucrit.for <- MCMCglmm(cbind(Mean.velo.BL.scale, Ucrit.BL.scale) ~ 1,
                            random = ~us(trait):Fish.ID,
                            prior = prior.multi, 
                            data = data.for2,
                            rcov = ~ us(trait):units, 
                            family = c("gaussian", "gaussian"), 
                            nitt = 400000, thin = 200, burnin = 1000, verbose = F)
summary(velo.ucrit.for)
```

```
## 
##  Iterations = 1001:399801
##  Thinning interval  = 200
##  Sample size  = 1995 
## 
##  DIC: 1538.906 
## 
##  G-structure:  ~us(trait):Fish.ID
## 
##                                                         post.mean   l-95% CI
## traitMean.velo.BL.scale:traitMean.velo.BL.scale.Fish.ID   0.41300  2.284e-01
## traitUcrit.BL.scale:traitMean.velo.BL.scale.Fish.ID       0.03004 -2.266e-02
## traitMean.velo.BL.scale:traitUcrit.BL.scale.Fish.ID       0.03004 -2.266e-02
## traitUcrit.BL.scale:traitUcrit.BL.scale.Fish.ID           0.01507  6.997e-09
##                                                         u-95% CI eff.samp
## traitMean.velo.BL.scale:traitMean.velo.BL.scale.Fish.ID  0.61277     1995
## traitUcrit.BL.scale:traitMean.velo.BL.scale.Fish.ID      0.11332     1995
## traitMean.velo.BL.scale:traitUcrit.BL.scale.Fish.ID      0.11332     1995
## traitUcrit.BL.scale:traitUcrit.BL.scale.Fish.ID          0.04938     1995
## 
##  R-structure:  ~us(trait):units
## 
##                                                       post.mean l-95% CI
## traitMean.velo.BL.scale:traitMean.velo.BL.scale.units   0.61819   0.5014
## traitUcrit.BL.scale:traitMean.velo.BL.scale.units      -0.03146  -0.1206
## traitMean.velo.BL.scale:traitUcrit.BL.scale.units      -0.03146  -0.1206
## traitUcrit.BL.scale:traitUcrit.BL.scale.units           1.00103   0.8371
##                                                       u-95% CI eff.samp
## traitMean.velo.BL.scale:traitMean.velo.BL.scale.units   0.7230     1795
## traitUcrit.BL.scale:traitMean.velo.BL.scale.units       0.0744     1995
## traitMean.velo.BL.scale:traitUcrit.BL.scale.units       0.0744     1995
## traitUcrit.BL.scale:traitUcrit.BL.scale.units           1.1614     1995
## 
##  Location effects: cbind(Mean.velo.BL.scale, Ucrit.BL.scale) ~ 1 
## 
##             post.mean  l-95% CI  u-95% CI eff.samp pMCMC
## (Intercept) -0.002666 -0.096315  0.103792     1870 0.946
```

Only for warm fish

```
hot.velo.ucrit.for <- MCMCglmm(cbind(Mean.velo.BL.scale, Ucrit.BL.scale) ~ 1,
                           random = ~us(trait):Fish.ID,
                           prior = prior.multi, 
                           data = data.for2[which(data.for2$Dev.temp == "Hot"),],
                           rcov = ~ us(trait):units, 
                           family = c("gaussian", "gaussian"), 
                           nitt = 400000, thin = 200, burnin = 1000, verbose = F)
summary(hot.velo.ucrit.for)
```

```
## 
##  Iterations = 1001:399801
##  Thinning interval  = 200
##  Sample size  = 1995 
## 
##  DIC: 816.176 
## 
##  G-structure:  ~us(trait):Fish.ID
## 
##                                                         post.mean   l-95% CI
## traitMean.velo.BL.scale:traitMean.velo.BL.scale.Fish.ID   0.37765  1.248e-01
## traitUcrit.BL.scale:traitMean.velo.BL.scale.Fish.ID       0.01413 -5.851e-02
## traitMean.velo.BL.scale:traitUcrit.BL.scale.Fish.ID       0.01413 -5.851e-02
## traitUcrit.BL.scale:traitUcrit.BL.scale.Fish.ID           0.02356  1.801e-09
##                                                         u-95% CI eff.samp
## traitMean.velo.BL.scale:traitMean.velo.BL.scale.Fish.ID  0.66269     1995
## traitUcrit.BL.scale:traitMean.velo.BL.scale.Fish.ID      0.10707     1995
## traitMean.velo.BL.scale:traitUcrit.BL.scale.Fish.ID      0.10707     1995
## traitUcrit.BL.scale:traitUcrit.BL.scale.Fish.ID          0.08657     1995
## 
##  R-structure:  ~us(trait):units
## 
##                                                       post.mean l-95% CI
## traitMean.velo.BL.scale:traitMean.velo.BL.scale.units   0.62217  0.46609
## traitUcrit.BL.scale:traitMean.velo.BL.scale.units       0.07473 -0.06122
## traitMean.velo.BL.scale:traitUcrit.BL.scale.units       0.07473 -0.06122
## traitUcrit.BL.scale:traitUcrit.BL.scale.units           1.11102  0.88589
##                                                       u-95% CI eff.samp
## traitMean.velo.BL.scale:traitMean.velo.BL.scale.units   0.7821     1995
## traitUcrit.BL.scale:traitMean.velo.BL.scale.units       0.2275     2164
## traitMean.velo.BL.scale:traitUcrit.BL.scale.units       0.2275     2164
## traitUcrit.BL.scale:traitUcrit.BL.scale.units           1.3740     1995
## 
##  Location effects: cbind(Mean.velo.BL.scale, Ucrit.BL.scale) ~ 1 
## 
##             post.mean l-95% CI u-95% CI eff.samp pMCMC
## (Intercept)   0.04948 -0.11359  0.19523     1995 0.536
```

Only for cold fish

```
cold.velo.ucrit.for <- MCMCglmm(cbind(Mean.velo.BL.scale, Ucrit.BL.scale) ~ 1,
                           random = ~us(trait):Fish.ID,
                           prior = prior.multi, 
                           data = data.for2[which(data.for2$Dev.temp == "Cold"),],
                           rcov = ~ us(trait):units, 
                           family = c("gaussian", "gaussian"), 
                           nitt = 400000, thin = 200, burnin = 1000, verbose = F)
summary(cold.velo.ucrit.for)
```

```
## 
##  Iterations = 1001:399801
##  Thinning interval  = 200
##  Sample size  = 1995 
## 
##  DIC: 726.9009 
## 
##  G-structure:  ~us(trait):Fish.ID
## 
##                                                         post.mean   l-95% CI
## traitMean.velo.BL.scale:traitMean.velo.BL.scale.Fish.ID   0.46550  1.755e-01
## traitUcrit.BL.scale:traitMean.velo.BL.scale.Fish.ID       0.03438 -5.691e-02
## traitMean.velo.BL.scale:traitUcrit.BL.scale.Fish.ID       0.03438 -5.691e-02
## traitUcrit.BL.scale:traitUcrit.BL.scale.Fish.ID           0.02901  5.477e-08
##                                                         u-95% CI eff.samp
## traitMean.velo.BL.scale:traitMean.velo.BL.scale.Fish.ID   0.8421     1995
## traitUcrit.BL.scale:traitMean.velo.BL.scale.Fish.ID       0.1547     1995
## traitMean.velo.BL.scale:traitUcrit.BL.scale.Fish.ID       0.1547     1995
## traitUcrit.BL.scale:traitUcrit.BL.scale.Fish.ID           0.1029     1995
## 
##  R-structure:  ~us(trait):units
## 
##                                                       post.mean l-95% CI
## traitMean.velo.BL.scale:traitMean.velo.BL.scale.units    0.6406   0.4804
## traitUcrit.BL.scale:traitMean.velo.BL.scale.units       -0.1476  -0.2904
## traitMean.velo.BL.scale:traitUcrit.BL.scale.units       -0.1476  -0.2904
## traitUcrit.BL.scale:traitUcrit.BL.scale.units            0.9345   0.7122
##                                                       u-95% CI eff.samp
## traitMean.velo.BL.scale:traitMean.velo.BL.scale.units  0.81280     1995
## traitUcrit.BL.scale:traitMean.velo.BL.scale.units     -0.01319     1855
## traitMean.velo.BL.scale:traitUcrit.BL.scale.units     -0.01319     1855
## traitUcrit.BL.scale:traitUcrit.BL.scale.units          1.15676     1995
## 
##  Location effects: cbind(Mean.velo.BL.scale, Ucrit.BL.scale) ~ 1 
## 
##             post.mean l-95% CI u-95% CI eff.samp pMCMC
## (Intercept)   -0.0384  -0.1894   0.1245     2015 0.614
```

No real difference between the two treatments

# BREADTH & MODE OF UCRIT

We estimated the breadth and mode of each individuals Ucrit curve Breadth: range of temperatures over which the animal maintained 80% max swimming Mode: Temperature at which animal achieved max swimming

```
max <- read.csv("Laskowski et al_Frontiers_breadth and mode_DATA.csv")
max.fem <- filter(max, Sex == 0)
```

## > Mode

two-way interaction

```
mode1 <- lm(Mode ~ Dev.temp*Species, data = max.fem)
summary(mode1)
```

```
## 
## Call:
## lm(formula = Mode ~ Dev.temp * Species, data = max.fem)
## 
## Residuals:
##      Min       1Q   Median       3Q      Max 
## -19.0239  -1.8031  -0.3918   1.3626  13.8461 
## 
## Coefficients:
##                             Estimate Std. Error t value Pr(>|t|)    
## (Intercept)                  32.3898     0.7158  45.248   <2e-16 ***
## Dev.tempHot                   0.7431     0.9692   0.767   0.4450    
## Speciesmexicana              -1.8331     1.0026  -1.828   0.0704 .  
## Dev.tempHot:Speciesmexicana   1.6855     1.4021   1.202   0.2321    
## ---
## Signif. codes:  0 '***' 0.001 '**' 0.01 '*' 0.05 '.' 0.1 ' ' 1
## 
## Residual standard error: 3.579 on 101 degrees of freedom
##   (3 observations deleted due to missingness)
## Multiple R-squared:  0.07919,    Adjusted R-squared:  0.05184 
## F-statistic: 2.895 on 3 and 101 DF,  p-value: 0.03889
```

No interaction, what about within each species?

Mexicana:

```
mode.mex <- lm(Mode ~ Dev.temp, data = max.fem[which(max.fem$Species == "mexicana"),])
summary(mode.mex)
```

```
## 
## Call:
## lm(formula = Mode ~ Dev.temp, data = max.fem[which(max.fem$Species == 
##     "mexicana"), ])
## 
## Residuals:
##     Min      1Q  Median      3Q     Max 
## -5.0046 -1.7789 -0.4648  1.3305 10.4144 
## 
## Coefficients:
##             Estimate Std. Error t value Pr(>|t|)    
## (Intercept)  30.5567     0.5383  56.764  < 2e-16 ***
## Dev.tempHot   2.4286     0.7770   3.126  0.00301 ** 
## ---
## Signif. codes:  0 '***' 0.001 '**' 0.01 '*' 0.05 '.' 0.1 ' ' 1
## 
## Residual standard error: 2.745 on 48 degrees of freedom
## Multiple R-squared:  0.1691, Adjusted R-squared:  0.1518 
## F-statistic:  9.77 on 1 and 48 DF,  p-value: 0.003009
```

```
mode.mex1 <- lm(Mode ~ 1, data = max.fem[which(max.fem$Species == "mexicana"),])
anova(mode.mex, mode.mex1)
```

```
## Analysis of Variance Table
## 
## Model 1: Mode ~ Dev.temp
## Model 2: Mode ~ 1
##   Res.Df    RSS Df Sum of Sq      F   Pr(>F)   
## 1     48 361.65                                
## 2     49 435.26 -1   -73.611 9.7701 0.003009 **
## ---
## Signif. codes:  0 '***' 0.001 '**' 0.01 '*' 0.05 '.' 0.1 ' ' 1
```

formosa:

```
mode.for <- lm(Mode ~ Dev.temp, data = max.fem[which(max.fem$Species == "formosa"),])
summary(mode.for)
```

```
## 
## Call:
## lm(formula = Mode ~ Dev.temp, data = max.fem[which(max.fem$Species == 
##     "formosa"), ])
## 
## Residuals:
##      Min       1Q   Median       3Q      Max 
## -19.0239  -2.0083  -0.2145   1.3820  13.8461 
## 
## Coefficients:
##             Estimate Std. Error t value Pr(>|t|)    
## (Intercept)  32.3898     0.8388  38.615   <2e-16 ***
## Dev.tempHot   0.7431     1.1357   0.654    0.516    
## ---
## Signif. codes:  0 '***' 0.001 '**' 0.01 '*' 0.05 '.' 0.1 ' ' 1
## 
## Residual standard error: 4.194 on 53 degrees of freedom
##   (3 observations deleted due to missingness)
## Multiple R-squared:  0.008013,   Adjusted R-squared:  -0.0107 
## F-statistic: 0.4281 on 1 and 53 DF,  p-value: 0.5157
```

```
mode.for1 <- lm(Mode ~ 1, data = max.fem[which(max.fem$Species == "formosa"),])
anova(mode.for, mode.for1)
```

```
## Analysis of Variance Table
## 
## Model 1: Mode ~ Dev.temp
## Model 2: Mode ~ 1
##   Res.Df    RSS Df Sum of Sq      F Pr(>F)
## 1     53 932.21                           
## 2     54 939.74 -1   -7.5304 0.4281 0.5157
```

## > Breadth

two-way interaction

```
breadth1 <- lm(Breadth ~ Dev.temp*Species, data = max.fem)
summary(breadth1)
```

```
## 
## Call:
## lm(formula = Breadth ~ Dev.temp * Species, data = max.fem)
## 
## Residuals:
##     Min      1Q  Median      3Q     Max 
## -6.3667 -2.8280 -0.9932  1.8670 17.6834 
## 
## Coefficients:
##                             Estimate Std. Error t value Pr(>|t|)    
## (Intercept)                  18.4404     0.8669  21.272   <2e-16 ***
## Dev.tempHot                   1.2274     1.1830   1.038    0.302    
## Speciesmexicana               0.5089     1.2141   0.419    0.676    
## Dev.tempHot:Speciesmexicana   2.6367     1.7044   1.547    0.125    
## ---
## Signif. codes:  0 '***' 0.001 '**' 0.01 '*' 0.05 '.' 0.1 ' ' 1
## 
## Residual standard error: 4.334 on 100 degrees of freedom
##   (4 observations deleted due to missingness)
## Multiple R-squared:  0.1305, Adjusted R-squared:  0.1044 
## F-statistic: 5.003 on 3 and 100 DF,  p-value: 0.002834
```

Mexicana:

```
breadth.mex <- lm(Breadth ~ Dev.temp, data = max.fem[which(max.fem$Species == "mexicana"),])
summary(breadth.mex)
```

```
## 
## Call:
## lm(formula = Breadth ~ Dev.temp, data = max.fem[which(max.fem$Species == 
##     "mexicana"), ])
## 
## Residuals:
##    Min     1Q Median     3Q    Max 
## -6.367 -2.483 -0.811  1.592 10.608 
## 
## Coefficients:
##             Estimate Std. Error t value Pr(>|t|)    
## (Intercept)  18.9493     0.7885  24.033  < 2e-16 ***
## Dev.tempHot   3.8641     1.1380   3.395  0.00138 ** 
## ---
## Signif. codes:  0 '***' 0.001 '**' 0.01 '*' 0.05 '.' 0.1 ' ' 1
## 
## Residual standard error: 4.02 on 48 degrees of freedom
## Multiple R-squared:  0.1937, Adjusted R-squared:  0.1769 
## F-statistic: 11.53 on 1 and 48 DF,  p-value: 0.001384
```

```
breadth.mex1 <- lm(Breadth ~ 1, data = max.fem[which(max.fem$Species == "mexicana"),])
anova(breadth.mex, breadth.mex1)
```

```
## Analysis of Variance Table
## 
## Model 1: Breadth ~ Dev.temp
## Model 2: Breadth ~ 1
##   Res.Df    RSS Df Sum of Sq      F   Pr(>F)   
## 1     48 775.84                                
## 2     49 962.18 -1   -186.34 11.529 0.001384 **
## ---
## Signif. codes:  0 '***' 0.001 '**' 0.01 '*' 0.05 '.' 0.1 ' ' 1
```

Formosa:

```
breadth.for <- lm(Breadth ~ Dev.temp, data = max.fem[which(max.fem$Species == "formosa"),])
summary(breadth.for)
```

```
## 
## Call:
## lm(formula = Breadth ~ Dev.temp, data = max.fem[which(max.fem$Species == 
##     "formosa"), ])
## 
## Residuals:
##    Min     1Q Median     3Q    Max 
## -5.951 -3.074 -1.134  2.084 17.683 
## 
## Coefficients:
##             Estimate Std. Error t value Pr(>|t|)    
## (Intercept)  18.4404     0.9211  20.020   <2e-16 ***
## Dev.tempHot   1.2274     1.2569   0.977    0.333    
## ---
## Signif. codes:  0 '***' 0.001 '**' 0.01 '*' 0.05 '.' 0.1 ' ' 1
## 
## Residual standard error: 4.605 on 52 degrees of freedom
##   (4 observations deleted due to missingness)
## Multiple R-squared:  0.01801,    Adjusted R-squared:  -0.0008761 
## F-statistic: 0.9536 on 1 and 52 DF,  p-value: 0.3333
```

```
breadth.for1 <- lm(Breadth ~ 1, data = max.fem[which(max.fem$Species == "formosa"),])
anova(breadth.for, breadth.for1)
```

```
## Analysis of Variance Table
## 
## Model 1: Breadth ~ Dev.temp
## Model 2: Breadth ~ 1
##   Res.Df    RSS Df Sum of Sq      F Pr(>F)
## 1     52 1103.0                           
## 2     53 1123.2 -1   -20.227 0.9536 0.3333
```

# FIGURES

## Fig 2

```
data <- read.csv("Both species_combined data_analysis.csv")
data.fem <- filter(data, Sex == 0)
data.fem <- group_by(data.fem, Species) %>% 
  mutate(Mean.velo.BL.scale = as.numeric(scale(Mean.velo.BL))) %>%
  mutate(Ucrit.BL.scale = as.numeric(scale(Ucrit.BL))) %>%
  mutate(Length.scale = as.numeric(scale(Length))) %>%
  mutate(Order.act = Test.day.act-3) %>%
  mutate(Order.swim = Test.day.swim-3) %>%
  mutate(Temp.cen = Test.temp-28)
data.for <- filter(data.fem, Species == "formosa")
data.mex <- filter(data.fem, Species == "mexicana")

summarySE <- function(data=NULL, measurevar, groupvars=NULL, na.rm=T,
                      conf.interval=.95, .drop=TRUE) {
  library(plyr)
  
  # New version of length which can handle NA's: if na.rm==T, don't count them
  length2 <- function (x, na.rm=T) {
    if (na.rm) sum(!is.na(x))
    else       length(x)
  }
  
  # This does the summary. For each group's data frame, return a vector with
  # N, mean, and sd
  datac <- ddply(data, groupvars, .drop=.drop,
                 .fun = function(xx, col) {
                   c(N    = length2(xx[[col]], na.rm=na.rm),
                     mean = mean   (xx[[col]], na.rm=na.rm),
                     sd   = sd     (xx[[col]], na.rm=na.rm)
                   )
                 },
                 measurevar
  )
  
  # Rename the "mean" column    
  #datac <- rename(datac, c("mean" = measurevar))
  
  datac$se <- datac$sd / sqrt(datac$N)  # Calculate standard error of the mean
  
  # Confidence interval multiplier for standard error
  # Calculate t-statistic for confidence interval: 
  # e.g., if conf.interval is .95, use .975 (above/below), and use df=N-1
  ciMult <- qt(conf.interval/2 + .5, datac$N-1)
  datac$ci <- datac$se * ciMult
  
  return(datac)
}

velo.sum.mex <- summarySE(data.mex, measurevar = "Mean.velo.BL", groupvars = c("Dev.temp", "Test.temp"))
ucrit.sum.mex <- summarySE(data.mex, measurevar = "Ucrit.BL", groupvars = c("Dev.temp", "Test.temp"))
pd <- position_dodge(width = 0.8)

# with smoothed lines 
swim.mex2 <- ggplot(ucrit.sum.mex, aes(x=Test.temp, y=mean, group=factor(Dev.temp))) + 
  geom_errorbar(aes(ymin=mean-ci, ymax=mean+ci), size = 1, width=.3, colour= "black", position = pd) +
  stat_smooth(aes(x = Test.temp, y = mean, color = factor(Dev.temp)),
              method = "lm", formula = y ~ poly(x, 2)) +
  geom_point(aes(fill = factor(Dev.temp)), position=pd, size=4.5, shape=21 ) +
  scale_colour_manual(name="Developmental\ntemperature",    # Legend label, use darker colors
                      breaks=c("Cold", "Hot"),
                      labels=c("Cold", "Hot"),
                      values = c("#0000FF", "#990000")) +  
  scale_fill_manual(name="Developmental\ntemperature",    # Legend label, use darker colors
                    breaks=c("Cold", "Hot"),
                    labels=c("Cold", "Hot"),
                    values = c("white", "#990000")) + 
  scale_x_continuous(breaks = c(18, 22, 28, 34, 38), labels = c(" ", " ", " ", " ", " ")) +
  scale_y_continuous(limits = c(2,12), breaks = c(2,4,6,8,10,12)) +
  annotate("text", x = 18, y = 12, label = "a", size = 6) +
  ylab("Maximum sustained swimming speed (body length/s)") + 
  xlab("") +
  theme_classic() +
  ggtitle("Atlantic mollies") +
  theme(plot.title = element_text(lineheight=1.2,  hjust = 0.5),
        legend.position = "none")

velo.mex <- ggplot(velo.sum.mex, aes(x=Test.temp, y=mean, group=factor(Dev.temp))) + 
  geom_errorbar(aes(ymin=mean-ci, ymax=mean+ci), size = 1, width=.3, colour= "black", position = pd) +
  geom_line(aes(colour= factor(Dev.temp)), size = 1) +
  geom_point(aes(fill = factor(Dev.temp)), position=pd, size=4.5, shape=21 ) +
  scale_colour_manual(name="Developmental\ntemperature",    # Legend label, use darker colors
                      breaks=c("Cold", "Hot"),
                      labels=c("Cold", "Hot"),
                      values = c("#0000FF", "#990000")) +  
  scale_fill_manual(name="Developmental\ntemperature",    # Legend label, use darker colors
                    breaks=c("Cold", "Hot"),
                    labels=c("Cold", "Hot"),
                    values = c("white", "#990000")) + 
  scale_x_continuous(breaks = c(18, 22, 28, 34, 38)) +
  scale_y_continuous(limits = c(0,2), breaks = c(0,0.5, 1, 1.5, 2) ) +
  annotate("text", x = 18, y = 2, label ="b", size = 6) +
  ylab("Mean velocity in an open field (body length/s)") + 
  xlab("Test temperature (?C)") +
  theme_classic() +
  theme(legend.position = "none")
  
velo.sum.for <- summarySE(data.for, measurevar = "Mean.velo.BL", groupvars = c("Dev.temp", "Test.temp"))
ucrit.sum.for <- summarySE(data.for, measurevar = "Ucrit.BL", groupvars = c("Dev.temp", "Test.temp"))
pd <- position_dodge(width = 0.8)

swim.for <- ggplot(ucrit.sum.for, aes(x=Test.temp, y=mean, group=factor(Dev.temp))) + 
  geom_errorbar(aes(ymin=mean-ci, ymax=mean+ci), size = 1, width=.3, colour= "black", position = pd) +
  stat_smooth(aes(x = Test.temp, y = mean, color = factor(Dev.temp)),
              method = "lm", formula = y ~ poly(x, 2)) +
  geom_point(aes(fill = factor(Dev.temp)), position=pd, size=4.5, shape=21 ) +
  scale_colour_manual(name="Developmental\ntemperature",    # Legend label, use darker colors
                      breaks=c("22", "28"),
                      labels=c("Cold", "Hot"),
                      values = c("#0000FF", "#990000")) +  
  scale_fill_manual(name="Developmental\ntemperature",    # Legend label, use darker colors
                    breaks=c("22", "28"),
                    labels=c("Cold", "Hot"),
                    values = c("white", "#990000")) + 
  scale_x_continuous(breaks = c(18, 22, 28, 34, 38), labels = c(" ", " ", " ", " ", " ")) +
  scale_y_continuous(limits = c(2,12), breaks = c(2,4,6,8,10,12), labels = c(" ", "", " ", " ", " ", " ")) +
  annotate("text", x = 18, y = 12, label = "c", size = 6) +
  ylab("") + 
  xlab("") +
  theme_classic() +
  ggtitle("Amazon mollies") +
  theme(plot.title = element_text(lineheight=1.2,  hjust = 0.5),
        legend.position = "none")


velo.for <- ggplot(velo.sum.for, aes(x=Test.temp, y=mean, group=factor(Dev.temp))) + 
  geom_errorbar(aes(ymin=mean-ci, ymax=mean+ci), size = 1, width=.3, colour= "black", position = pd) +
  geom_line(aes(colour= factor(Dev.temp)), size = 1) +
  geom_point(aes(fill = factor(Dev.temp)), position=pd, size=4.5, shape=21 ) +
  scale_colour_manual(name="Developmental\ntemperature",    # Legend label, use darker colors
                      breaks=c("22", "28"),
                      labels=c("Cold", "Hot"),
                      values = c("#0000FF", "#990000")) +  
  scale_fill_manual(name="Developmental\ntemperature",    # Legend label, use darker colors
                    breaks=c("22", "28"),
                    labels=c("Cold", "Hot"),
                    values = c("white", "#990000")) + 
  scale_x_continuous(breaks = c(18, 22, 28, 34, 38)) +
  scale_y_continuous(limits = c(0,2), breaks = c(0,0.5,1,1.5,2), labels = c(" ", " ", " ", " ", " ")) +
  annotate("text", x = 18, y = 2.0, label ="d", size = 6) +
  ylab("") + 
  xlab("Test temperature (?C)") +
  theme_classic() +
  theme(plot.title = element_text(lineheight=1,  hjust = 0.5)) +
  theme(legend.position = c(0.2, 0.2))

library(gridExtra)

both.plot2 <- arrangeGrob(swim.mex2, swim.for,velo.mex,  velo.for,   ncol = 2)
both.plot2
```

```
## TableGrob (2 x 2) "arrange": 4 grobs
##   z     cells    name           grob
## 1 1 (1-1,1-1) arrange gtable[layout]
## 2 2 (1-1,2-2) arrange gtable[layout]
## 3 3 (2-2,1-1) arrange gtable[layout]
## 4 4 (2-2,2-2) arrange gtable[layout]
```

```
#ggsave("Both species behavior_2.tiff", plot = both.plot2, height = 24, width = 24, units = "cm", dpi = 300)
```

## Fig 3

```
mode.graph <- ggplot(max.fem, aes(x = Species, y = Mode, fill = Dev.temp)) +
  geom_boxplot(aes(fill = Dev.temp), size = 1.2) + 
  scale_fill_manual(name="Developmental\ntemperature", values = c("white", "#990000")) +
  scale_color_manual(name="Developmental\ntemperature", values = c("#0000FF", "black")) +
  scale_y_continuous(limits = c(20, 44), breaks = c(20,24,28,32,36,40,44)) +
  scale_x_discrete(labels =c("Amazon", "Atlantic")) +
  geom_segment(aes(x = 1.8, y = 44, xend = 2.2, yend = 44), size = 1.2) +
  annotate("text", x = 2, y = 42.5, label = "*", size = 12) + 
  annotate("text", x = 0.5, y = 44, label = "a", size = 6) +
  labs(y=expression("Mode U"[crit]*" (?C)")) +
  xlab("") +
  theme_classic() +
  theme(axis.text.y =element_text(size=10),
        axis.text.x = element_text(size = 12),
        axis.title=element_text(size=14,face="bold"),
        legend.position = c(0.2, 0.15))

breadth.graph <- ggplot(max.fem, aes(x = Species, y = Breadth, fill = Dev.temp)) +
  geom_boxplot(aes(fill = Dev.temp), size = 1) + 
  scale_fill_manual(values = c("white", "#990000")) +
  scale_color_manual(values = c("#0000FF", "black")) +
  scale_y_continuous(limits = c(10, 34), breaks = c(10,14,18,22,26,30,34)) +
  scale_x_discrete(labels =c("Amazon", "Atlantic")) +
  geom_segment(aes(x = 1.8, y = 34, xend = 2.2, yend = 34), size = 1.2) +
  annotate("text", x = 2, y = 32.5, label = "*", size = 12) + 
  annotate("text", x = 0.5, y = 34, label = "b", size = 6) +
  labs(y=expression("Breadth U"[crit]*" (?C)")) +
  xlab("") + 
  theme_classic() +
  theme(axis.text.y =element_text(size=10),
        axis.text.x = element_text(size = 12),
        axis.title=element_text(size=14,face="bold"),
        legend.position = "none")


both.plot3 <- arrangeGrob(mode.graph, breadth.graph, ncol = 2)
```

```
## Warning: Removed 5 rows containing non-finite values (stat_boxplot).

## Warning: Removed 5 rows containing non-finite values (stat_boxplot).
```

```
both.plot3
```

```
## TableGrob (1 x 2) "arrange": 2 grobs
##   z     cells    name           grob
## 1 1 (1-1,1-1) arrange gtable[layout]
## 2 2 (1-1,2-2) arrange gtable[layout]
```

```
#ggsave("Ucrit mode and breadth.tiff", plot = both.plot3, height = 14, width = 20, units = "cm", dpi = 300)
```

Add a new chunk by clicking the *Insert Chunk* button on the toolbar or by pressing *Ctrl+Alt+I*.

When you save the notebook, an HTML file containing the code and output will be saved alongside it (click the *Preview* button or press *Ctrl+Shift+K* to preview the HTML file).

The preview shows you a rendered HTML copy of the contents of the editor. Consequently, unlike *Knit*, *Preview* does not run any R code chunks. Instead, the output of the chunk when it was last run in the editor is displayed.
